# Accurate bladder cancer diagnosis using ensemble deep leaning

**DOI:** 10.1038/s41598-025-95002-0

**Published:** 2025-04-15

**Authors:** Rana A. El-Atier, M. S. Saraya, Ahmed I. Saleh, Asmaa H. Rabie

**Affiliations:** Computers and Control Department, Faculty of Engineering Department, Faculty of Engineering, Mansoura University, Mansoura, 35516 Egypt

**Keywords:** Deep learning, Bladder cancer, Ensemble classification, Diagnosis, Voting, Diseases, Health care, Engineering

## Abstract

There are an estimated 1.3 million cases of cancer globally each year, making it one of the most serious types of urinary tract cancer. The methods used today for diagnosing and monitoring bladder cancer are intrusive, costly, and time-consuming. In clinical practice, invasive biopsy followed by histological examination continues to be the gold standard for diagnosis. Bladder cancer biomarkers have been used in expensive diagnostic tests created recently, however their reliability is limited by their high rates of false positives and false negatives. The potential and use of artificial intelligence in urological diseases have been the subject of several research, as interest in artificial intelligence in medicine has grown recently. In this paper, a new bladder cancer model called Ensemble Deep Learning (EDL) will be provided to accurately diagnose patients. Outlier rejection is used to filter data using the interquartile range (IQR) then the image diagnosis. The proposed EDL consists of three deep learning algorithms, which are; Convolutional Neural Network (CNN), Generative Adversarial Network (GAN), and a new deep learning method called Explainable Deep Learning (XDL) that depends on Guided Gradient Weighted Class Activation Map (Guided Grad-CAM). In fact, Guided Grad-CAM enables doctor to understand the diagnose. A new voting mechanism will be used to integrate the results of all three methods to produce the final result to accurately diagnose bladder cancer cases. In fact, the used voting method depends on using majority voting based on two different scenarios according to the results of CNN, GAN, and XDL. If these three methods give the same class category, then the final diagnosis will be this class category. On the other hand, if the three methods give different class category, then the final result will be followed by the accuracy of each class. The proposed EDL model was tested after several trials. The results have proved that EDL model is more efficient and more accurate to diagnose bladder cancer disease. It introduced the highest accuracy results and the lowest error results as well as execution time.

## Introduction

Bladder cancer is one of the most prevalent cancers worldwide, responsible for approximately 213,000 deaths and 573,000 new cases in 2020^1^. A major global research study on patients with suspected urinary tract infections, IDENTIFY, found that bladder cancer is the most common cancer diagnosis among patients with hematuria^2^. Patients with muscle-invasive bladder cancer have a significantly lower five-year survival rate when the tumor spreads to other layers of the bladder^3^. In contrast, around 90% of individuals with non-muscle invasive bladder tumors are expected to survive for five years. The IDENTIFY study recently introduced a multivariable prediction model for diagnosing urinary tract tumors, which aids doctors in assessing patient risk early by identifying those with hematuria^4^. Despite advancements in surgery, diagnostics, and therapeutic approaches, the clinical outcomes for bladder cancer have not significantly improved over the past 30 years^5^. Accurate and consistently effective pathological evaluation techniques are crucial, as reducing bladder cancer mortality relies on early detection, precise diagnosis, and appropriate treatment interventions.

Right now, the gold standard for diagnosing and monitoring bladder cancer is white-light cystoscopy^6^. Patients undergo transurethral resection of the bladder tumor (TURBT) if abnormal tissue is discovered. During this procedure, an endoscope is inserted into the urinary tract, and any visible tumor lesions are removed. Based on their invasive potential, urothelial carcinoma is classified by the World Health Organization (WHO) into two main categories: high-grade carcinoma and low-grade carcinoma^7^. Visually classifying bladder cancer is a difficult task. Sometimes, lesions from high-grade and low-grade tumors appear quite similar, and there is a significant challenge in distinguishing between non-tumorous lesions and healthy tissue^8^. In actuality, histological examination of the removed tissue is necessary for a definitive cancer diagnosis, staging, and grading^9^. Notably, white-light cystoscopy fails to detect 20% of lesions, especially those classified as non-papillary or multi-focal, which contributes to increased morbidity and mortality rates^10^.

Artificial intelligence (AI) strategies, such as Deep Learning (DL) and Machine Learning (ML) algorithms^11,12^, are extensively employed in the diagnosis and prognosis of many illnesses, particularly those whose diagnoses rely on signal analysis or imaging^13,14^. Additionally, artificial intelligence can assist in identifying environmental or demographic factors that contribute to disease susceptibility or high-risk behaviors^15,16^. Using ML, computers can perform tasks typically carried out by medical experts. DL is a widely used ML subfield for medical image recognition. It is an advanced ML algorithm that constructs a deep architecture with multiple processing layers, stacking simple concepts on top of each other. In essence, DL represents the evolution of ML for large-scale data analysis^17,18^. Unlike traditional methods, which require human expertise to define patterns for classification, DL replaces this manual process with an automated approach that enables machines to extract and prioritize the most significant features through dataset training^8,19^.

It is well known that deep learning (DL) has gained scalability and success in recent years due to its ability to process large amounts of data efficiently and improve computational capacity. In various visual identification tasks, DL has significantly outperformed earlier advanced techniques. The largest object identification competition, known as the ImageNet Large Scale Visual Recognition Challenge (ILSVRC), is held annually^20^. Using a CNN model based on DL, academics were able to classify 1.3 million high-resolution images^21^. CNNs applied in cancer diagnosis have demonstrated excellent performance, often matching that of human specialists^20^. After receiving imaging data (such as lines, curves, and different colors), the algorithm’s initial layers are trained to recognize low-level features, while the higher-level layers produce diagnostic predictions^22^.

Artificial intelligence (AI) applications are increasingly enhancing the potential for diagnosing bladder cancer. AI tools for bladder cancer diagnosis integrate imaging with cystoscopy-based tumor identification, tumor staging, and tumor grading^23^. Specifically, AI subsets, including machine learning (ML) and deep learning (DL) approaches, have been extensively studied for their role in the diagnosis, prognosis, and outcome prediction of bladder cancer cases^23^.

## Research motivation

The motivation for using deep learning in bladder cancer diagnosis stems from the limitations of current diagnostic methods. Traditional approaches, such as cystoscopy and biopsy, are invasive, uncomfortable for patients, and can suffer from inter-observer variability. Furthermore, these methods might miss small or subtly different cancerous lesions, delaying diagnosis and potentially impacting patient outcomes. Deep learning, with its ability to automatically analyze complex medical images like cystoscopy videos or histopathology slides, offers a non-invasive or minimally invasive alternative or adjunct. Deep learning models can possibly lead to earlier and more accurate diagnosis by learning to recognize complex patterns and traits indicative of bladder cancer that may be invisible to the human eye by training on large datasets. Personalized medicine, in which treatment plans are customized to each patient’s risk profile, has the potential to improve bladder cancer patients’ quality of life and survival rates. This is made possible by the enhanced diagnostic capabilities. Deep learning-based automated analysis can also improve clinical process efficiency and lessen the workload for radiologists and pathologists.

### The contribution of the paper

The main contribution of the paper is summarized as follow:

- Outlier rejection was applied to filter images using VGG16 and IQR before being fed into the EDL model.
- Ensemble Deep Learning (EDL) has been introduced to diagnose bladder cancer.
- EDL combines results of three models namely CNN, GAN, and XDL to obtain accurate result using new voting strategy.
- Hence, the decision of the proposed EDL is provided by using relative majority voting method to combine between three models by using one of two scenarios.
- The first scenario is related to the case where the same category is selected by many models and then this category will be followed as a final result.
- The second scenario concerns the case where the three models give different class category, thus, accuracy of each class will be used to reveal the final result.
- Experiments have demonstrated that EDL outperforms other models based on accuracy, precision, recall, error, and execution time. EDL provided the maximum accuracy, precision, recall, micro average recall and precision, macro average recall and precision, and F1-measure and also it provided the minimum timing of execution and error.

### The organization of the paper

This paper is organized in the following ways; Section “Related works” presents the related works about bladder cancer techniques using deep learning. Section “The proposed ensemble deep learning (EDL) model” discusses the proposed Ensemble Deep Learning (EDL) model in details and the experimental results are presented in Section “Simulation and results”. Section “Pros and cons” presents Pros and Cons for EDL. Finally, conclusions and future works are presented in Section “Conclusions and future works”.

## Related works

This section reviews previous research efforts on artificial intelligence methods and deep learning models for bladder cancer diagnosis. In^24^, the Hybrid Augmentation and Classification Model (HACM) was proposed to enhance precise lesion localization, delineation, and classification in bladder tumor transurethral resection, aiming to reduce the risk of cancer recurrence. HACM consists of three main components. The first component is an improved texture-constrained version of a conditional generative adversarial network for data augmentation. The second component is a novel mask sub-net scheme called the Multiple Mask and Boundary Scoring Region-based Convolutional Neural Network (R-CNN). It utilizes a new scoring module to refine object boundaries and improve segmentation accuracy by generating multiple masks from different levels of the mask sub-net pipeline. The third component is an accelerated training approach supported by the stochastic gradient descent optimizer with second momentum. HACM enhances data generation methods to improve accuracy. However, HACM requires validation and approval by medical professionals for clinical application.

According to^25^, the Classifying Urothelial Cancer Model (CUCM) was introduced using deep learning with a Convolutional Neural Network (CNN) to extract features from images by optimizing hyper parameters to achieve the highest grading accuracy. The model classifies urothelial carcinomas into low and high grades and also utilizes Grad-CAM for visualization, which was converted into a class activation map. This suggests that such a model may be employed as an additional diagnostic tool for urothelial carcinoma grading. Upon hyper parameter adjustment, the model demonstrated robustness with an overall accuracy of 90%. However, in this proposed method, it was unclear for doctors how exactly this algorithm arrives at the final judgment.

Related to^26^, the Imaging Examination Deep Learning (IEDL) model was proposed based on the U-Net neural network, incorporating the encoder along with the ResBlock in ResNet and the Dense Block in DenseNet. This technique was commonly used in the medical profession and allowed training to maintain the training set while reducing the total identification operation time. It used 2D CT images with a CNN, but 3D images provided more accuracy for doctors.

In^27^, the Improving Survival Prediction Model (ISPM) was proposed to develop an approach that capitalizes on the advantages of clinical data, deep learning, and radiomics. A nomogram was used to analyze the clinical data, while radiomics and deep learning models were used to analyze image data. To predict survival, the descriptors were input into a Back-Propagation Neural Network (BPNN) model. Patients with bladder cancer were reliably predicted to survive their cystectomy with this strategy. Despite these benefits, larger datasets were needed to achieve better results.

As presented in^28^, the Hybrid Bladder Cancer Classification (HBCC) model was proposed as a hybrid framework that combines statistical machine learning methods for classification with pre-trained deep neural networks for feature extraction. Radiologists could identify bladder cancer more precisely by using genomics to aid in the interpretation of CT scans. However, there were certain limitations with this approach. Because of its retrospective design and small, single-center dataset, the model’s diagnostic effectiveness may have been overstated.

In^29^, the Diagnose Bladder Cancer Model (DBCM) was introduced as a deep learning method for bladder cancer segmentation and detection that combines CNNs with a lightweight transformer without positional encoding and dual attention gates that combine spatial and self-attention to improve features. This model achieved a balance between computational efficiency and diagnostic accuracy in cystoscopic imaging. However, larger datasets were needed to achieve better results.

According to^30^, the Hybrid Deep Learning Diagnosis (HDLD) model was put out to examine deep learning architectures that were trained on a dataset of poorly labeled cystoscopy pictures, including U-Net++-VGG19, U-Net-VGG11, and FPN-ResNet34. The models shown potential and might be used in clinical settings without altering current treatment procedures. Lower-quality photos were used to train these models. These novel bladder cancer diagnostic techniques are contrasted in Table 1.

**Table 1:** A comparison between the bladder cancer techniques.

Technique	Year of publication	Advantages	Disadvantages	Model accuracy
The Hybrid Augmentation and Classification Model (HACM)^24^	2024	HACM is an accurate method Enhanced data augmentation Robustness to Variations	It does not generate enough different training data, which is essential for enhancing the generalization capabilities of the system Computational Complexity Need for Large Datasets (even with augmentation)	Reached 78%
Classifying Urothelial Cancer Model (CUCM)^25^	2023	CUCM is appropriate, dependable, and highly effective Objective and Quantitative Analysis	It is unclear for doctor how exactly such an algorithm arrives at the final judgment It is high Computational Cost	Reached 81%
Imaging Examination Deep Learning (IEDL) model^26^	2023	IEDL is an accurate method It has Efficient Feature Learning Potential for Real-time Applications	It is used CT images (2D) CNN but 3D images give more accurate for doctors It is Class Imbalance It is high Computational Cost	Reached 84%
Improving Survival Prediction Model (ISPM)^27^	2023	ISPM provides high accuracy It is Identification of Imaging Biomarkers Comprehensive Patient Assessment	The larger datasets are needed, to Achieving better results It is not Generalized for different data set	Reached 86%
Hybrid Bladder Cancer Classification (HBCC) model^28^	2023	HBCC provides accurate results Reduced Pathologist Workload	Its retrospective design and tiny, single-center dataset basis, the model’s diagnostic effectiveness may be overstated It need for Pathologist Validation	Reached 88%
Diagnose Bladder Cancer Model (DBCM)^29^	2024	DBCM is an accurate method Reduced Inter-observer Variability It make Early Detection	The larger datasets are needed, to Achieving better results It Need for Clinical Validation It has overfitting	Reached 90%
Hybrid Deep Leaning Diagnosis (HDLD) model^30^	2024	HDLD provides accurate results It Reduce Inter-observer Variability It Improve Risk Stratification	These models were trained using images of lower quality It is high Computational Cost It need for Clinical Validation	Reached 93%
Hyper classification and segmentation bladder cancer model [CSBCM]^31^	2024	CSBCM provides accurate results It produces more objective and consistent findings than human interpretation	CSBCM require lar and high quality datasets for training A result of this lack of openness, it may be difficult to trust and evaluate the findings	Reached 91%
Improving Detect Bladder Cancer[IDBC]^32^	2025	IDBC is a non-invasive procedure IDBC is an accurate method	IDBC requires specialized equipment and expertise IDBC instruments and the associated analysis can be expensive	Reached 92%
Bladder cancer segmentation model [BCSM]^33^	2024	BCSM reducing the time and effort BCSM is an accurate method	• Need for Large Datasets • It must. Extensive validation methods are required to avoid overfitting	Reached 93%
Developing algorithm in bladder cancer [DABC]^34^	2024	DABC has early detection IDBC is an accurate model	It required further research and validation before they can be widely It is a lengthy and costly process	Reached 94%
Bladder Cancer Diagnosis Using Ensemble Deep Leaning (EDL) Model		EDL is an accurate method Explainability Robustness	Its is Complexity Not fast enough	Reached 97%

In^35^, the Hyper Classification and Segmentation Bladder Cancer Model (CSBCM) was suggested as a way to classify and segment pictures from a cystoscope in order to identify bladder cancer. It made use of the deep learning models DeepLab v3+ and VGG19. VGG19 was utilized in this model along with an additional fully linked layer. The loss function was sparse categorical cross-entropy, and the classification model for bladder cancer lesions was developed with pathological data annotated. Additionally, the DeepLab v3+ model was used to segment each morphological kind of bladder cancer in the cystoscope pictures using the Dice coefficient loss function. Larger datasets were necessary to achieve better results despite these benefits.

According to^36^, the Improving Detection of Bladder Cancer (IDBC) model was presented as a system that compares cancerous cells with nonmalignant controls using genetically pure human bladder epithelial cell lines, demonstrating the great accuracy of the AFM approach in identifying bladder cancer cells. The model proved its efficacy by analyzing AFM adhesion maps using machine learning approaches in compliance with industry standards. The IDBC, on the other hand, required the approval and inclusion of medical experts in the clinical environment.

As presented in^37^, the Bladder Cancer Segmentation Model (BCSM) was proposed as a hyper-loss function model. The purpose of this work was to evaluate three deep learning (DL) models’ tumor segmentation abilities using multi-parametric MRI (mp-MRI) data. BCSM was able to detect bladder cancers on MRI images with more accuracy than conventional techniques. This improved bladder cancer segmentation on mp-MRI, leading to more accurate diagnosis, treatment planning, and potentially better patient outcomes. However, models trained on particular datasets might not generalize well to data from various scanners, organizations, or patient groups.

In^38^, the Developing Algorithm in Bladder Cancer (DABC) was put out to present a non-invasive, straightforward, fast, and precise test-based approach that may be extensively applied in clinical practice for the early identification and monitoring of bladder cancer. The goal of this research was to identify universal indicators in urine. This strategy lessens the financial and social strain on the country’s healthcare system, but its successful implementation in clinical settings depends on resolving its issues.

In our work to solve the problems associated with recent techniques from previous research, we proposed the Ensemble Deep Learning (EDL) model to diagnose bladder cancer. Outlier rejection method filter images using IQR^39^ and enter to EDL model. EDL combines three models, namely CNN, GAN, and XDL, to obtain accurate results. Compared to our proposal, it gives good results in feature extraction that enhance image quality. It provides a good degree of explainability, allowing doctors to understand which image regions contributed most to the diagnosis. Combining different models increases the power of prediction. If one model makes a mistake, the other models can compensate for the error, resulting in a more reliable overall prediction. EDL provides automated diagnosis, reducing the pathologist’s workload and improving diagnostic speed. Combining CNN, GAN, and XDL with Guided Grad-CAM and a specific voting mechanism represents a novel approach that may offer advantages over existing methods. However, EDL is not fast enough.

## The proposed ensemble deep learning (EDL) model

**Fig. 1:**
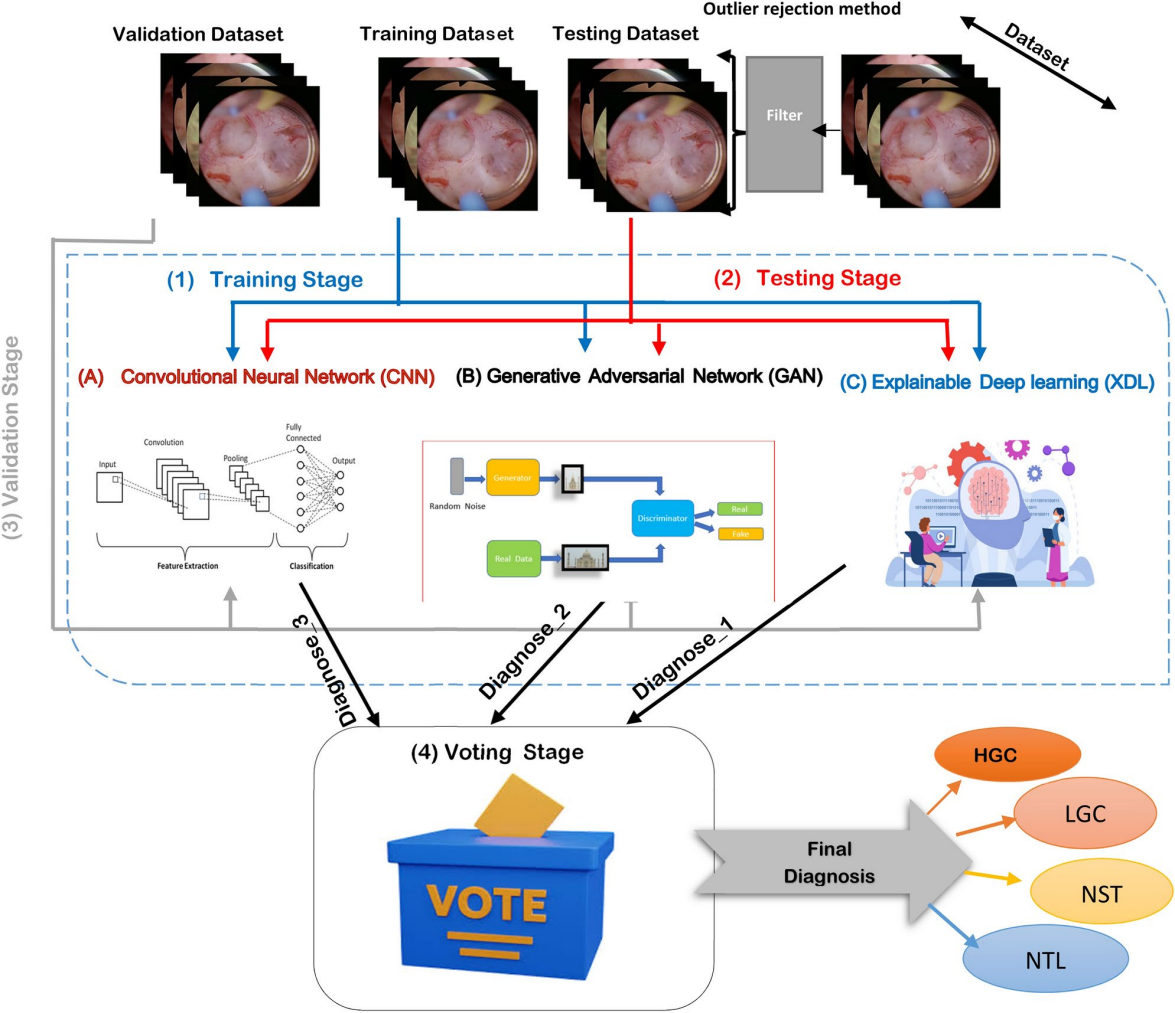
Ensemble deep learning (EDL) model.

Ensemble deep learning refers to the practice of combining multiple deep learning models to improve performance, robustness, and generalization. This method draws inspiration from ensemble learning in traditional machine learning, where combining multiple models often produces more effective results than using individual models alone. In this work, Outlier rejection is used to apply filters to data and verify their authenticity. And three diagnostic models are combined in the Ensemble Deep Learning (EDL) model to provide the most accurate diagnosis, as shown in Fig. 1. The first model is a Convolutional Neural Network (CNN)^40^, the second is a Generative Adversarial Network (GAN)^41^, and the third is a new deep learning method called Explainable Deep Learning (XDL).

In fact, CNNs have made significant strides in the medical field. They are highly effective in automatically identifying pertinent characteristics from X-rays, Magnetic Resonance Imaging (MRI), Computed Tomography (CT) scans, and other medical images without manual intervention. This capability helps in identifying patterns indicative of diseases or abnormalities. CNNs can automate certain tasks in the examination of medical images, such as tumor detection, organ segmentation, and disease diagnosis, reducing the time and effort required by medical professionals^41^. Their ability to analyze medical images aids in the early diagnosis of diseases by detecting subtle signs or markers that might not be obvious to human viewers right away. Unlike humans, CNNs do not suffer from fatigue or inconsistency, leading to more consistent and reproducible results in medical image analysis^42^. However, a large amount of labeled data is needed for CNN training, might be difficult to get in the medical industry because of privacy issues, data scarcity, and the requirement for professional annotation.

According to GANs, they can generate synthetic medical images or data, which can be used to augment limited datasets. This is especially helpful for medical imaging, since getting big, labeled datasets can be difficult due to privacy issues and the rarity of certain conditions^43^. GANs can be trained to identify anomalies or abnormalities in medical images, aiding in the early detection of diseases such as cancer or in identifying abnormalities in medical scans. GANs can also be used to enhance the resolution and quality of medical images, making it easier for clinicians to interpret and diagnose conditions accurately.

However, GANs are highly dependent on the quality and diversity of the training data^43^. Biases or errors in the training data can lead to biased or inaccurate generated outputs, potentially resulting in incorrect diagnoses or recommendations. Generated outputs from GANs are often considered "black-box" models, meaning they can be challenging to understand in terms of how and why a particular output was generated. This lack of interpretability can hinder trust and adoption in clinical settings, where transparency and interpretability are crucial^43^. GAN training takes a lot of time and computer power, particularly when working with large datasets or complex medical images. This can be a barrier for smaller healthcare institutions or research labs with limited resources.

On the other hand, XDL refers to techniques and models that aim to provide explanations or justifications for the predictions produced by deep learning models^44^. These techniques can be advantageous when analyzing medical images such as X-rays, MRIs, or CT scans. XDL methods offer explanations by highlighting regions of interest within medical images. For example, in a chest X-ray, the model might explain its diagnosis by indicating areas where abnormalities, such as nodules or infiltrates, are present^44^. This interpretability helps clinicians understand why the model made a particular diagnosis, enhancing trust and acceptance. Moreover, XDL techniques can generate explanations that align with ground truth annotations provided by radiologists or other medical experts. By highlighting areas of agreement between the model’s predictions and human annotations, XDL facilitates clinical validation and ensures that AI-driven diagnoses are consistent with established medical knowledge.

This combination is unique as it leverages each architecture’s advantages. Outlier rejection filter images from dataset, CNNs excel at extracting features from images, GANs can generate synthetic data or improve image quality when applied properly, and the suggested XDL with Guided Grad-CAM enhances explainability. Integrating these various methods represents a significant innovation. For medical applications, Guided Grad-CAM enables the visual explanation of the model’s diagnostic decisions. Compared to traditional "black-box" deep learning models, this explainability is a significant benefit because it fosters confidence and enables medical professionals to comprehend the rationale behind the diagnosis. This particular use of Guided Grad-CAM in a diagnostic model for bladder cancer appears to be unique.

- First scenario (Consensus): The diagnosis is clear if all three models concur.
- Second scenario (Disagreement): If the models disagree, the final diagnosis is made using the prediction accuracy of each model separately. When there is disagreement, this weighted voting method, based on individual model accuracy, is an innovative technique. Vote counting is not the only aspect; another factor is evaluating each "voter’s" credibility.

Medical images often contain complex patterns and structures that may be difficult to interpret, even with XDL explanations. For example, subtle features in an MRI scan could be missed by the model’s explanations, leading to potential misinterpretations by clinicians. Balancing the complexity of image features with the simplicity of explanations remains a challenge in XDL. XDL explanations may be subjective and influenced by factors such as the choice of algorithm or the interpretation of results by different individuals. In the case of medical images, different clinicians might have varying opinions on the significance of certain features highlighted by the model’s explanations, leading to inconsistencies in diagnosis or treatment decisions^44^.

From the above descriptions of CNN, GAN, and XDL, EDL combines the main characteristics of these three models to produce accurate results. In EDL, each of the three methods is trained in a parallel manner on the same training dataset. This diversity helps capture complementary information and reduces the risk of overfitting, resulting in improved accuracy in medical image analysis tasks such as segmentation, classification, and detection of abnormalities. In medical imaging, different imaging techniques (e.g., MRI, PET, CT) often provide complementary information about the underlying pathology. Ensemble learning integrates predictions from models trained on different modalities, leveraging the strengths of each modality to improve diagnostic accuracy and clinical decision-making.

This work focuses on the issue of classifying bladder material in domain-specific images obtained from TURBT procedures. It is important to keep in mind that annotations are available only in one of these images. Given that the majority of advanced computer vision techniques are sensitive to changes in domain^45^, we input images into ensemble deep learning models, train three models separately, and combine them using a relative majority voting method to integrate the three individual classifiers.

### Outlier rejection method

**Fig. 2:**
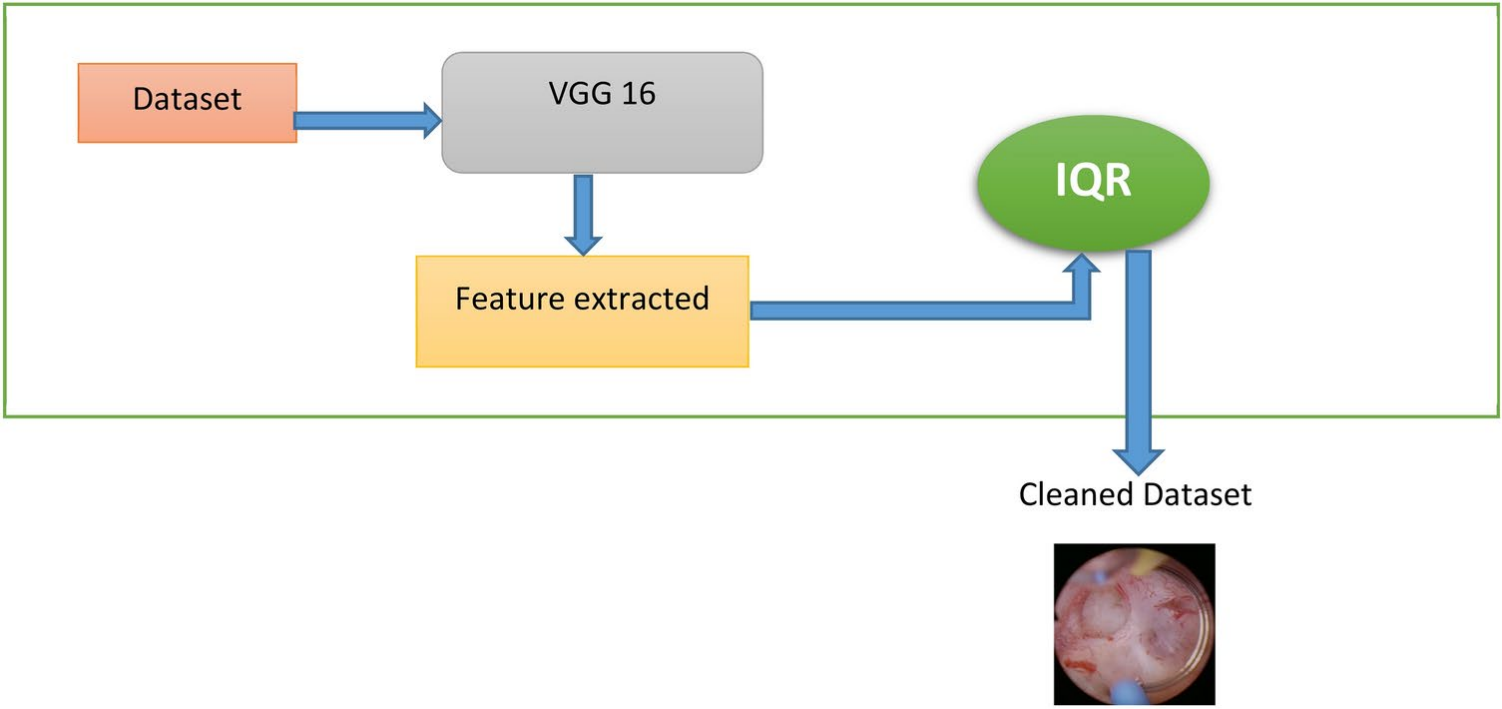
Outlier rejection method.

To improve the effectiveness of diagnosis and classification, I employed Outlier Rejection to filter the dataset, guaranteeing the validity of the photos and eliminating erroneous ones. We used VGG16 for feature extraction in this procedure, taking advantage of its deep layers to provide unique representations for every image. The Interquartile Range (IQR)^39^ approach was then used to identify and eliminate outliers as represented in Fig. 2. By enhancing the quality of the classification model’s input data, this method contributes to more accurate and consistent performance.

One statistical technique for identifying and eliminating outliers in data is the Interquartile Range (IQR). Pixel intensity, brightness, contrast, and color values are examples of numerical characteristics that may be derived from pictures and subjected to IQR in image processing. This technique enhances the quality and validity of picture databases by removing aberrant images.

We first extract a numerical characteristic, such the average brightness, from each image before using IQR to image processing. Next, we determine the data’s 25th and 75th percentiles, or the first quartile (QI1) and third quartile (QI3). The IQR is calculated as follows:

$$IQR = QI3 - QI1$$

This allows us to specify the range of values that are acceptable:

$$Lower\;Bound = QI1 - 1.5 \times IQR$$

$$Upper\;Bound = QI3 + 1.5 \times IQR$$

Images that have feature values outside of this range are rejected because they are deemed outliers. Computer vision, AI model training, and picture quality evaluation all make extensive use of this method. For instance, IQR may assist in eliminating overexposed or underexposed photographs from a collection of images, guaranteeing that only excellent, realistic-looking images are utilized. IQR is a useful tool in image processing and artificial intelligence applications since it can be used to improve picture collections, detect altered photos, and increase the performance of deep learning models.

### Convolutional neural network (CNN)

**Fig. 3:**
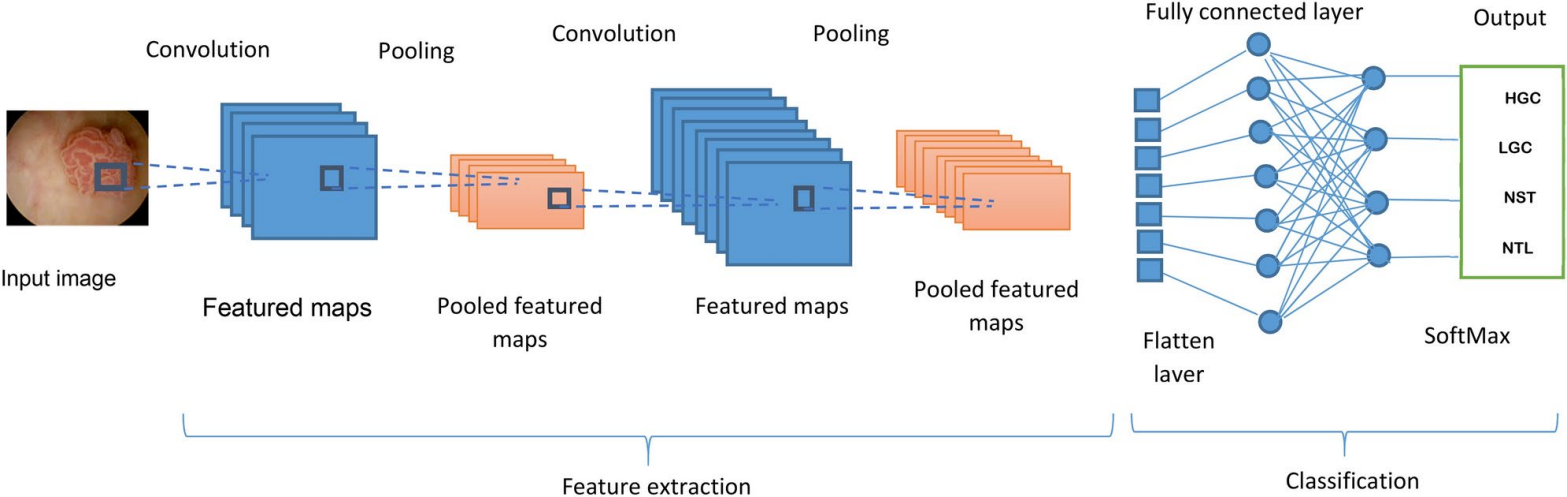
Bladder cancer model using CNN.

In a multilayer perceptron, there is a foundation for the Convolutional Neural Network (CNN), which is designed to analyze data in two dimensions. Because CNN has a high network depth and is frequently used with image data, it falls under the category of deep neural networks^46^. CNN’s architecture is similar to that of other neural networks in that each of its neurons has three functions: weight, bias, and activation. The CNN design is represented in Fig. 3^47^, where it consists of three layers: a convolution layer with Rectified Linear Unit (ReLU) activation and a pooling layer for feature extraction, in addition to a fully connected layer with softmax activation as the classification layer^40^.

According to the convolution layer, which serves as an input system model, it is the initial layer that processes the image. The input image, also known as the feature map, undergoes feature extraction by being convolved with a filter. According to the ReLU activation function, it has the advantage of decreasing errors by prolonging the neural network’s training process. When a pixel value is less than zero, ReLU sets all activation values to zero using (1)^47^. According to pooling layers, they are typically added periodically after several convolution layers. The pooling layer has a number of benefits, including the ability to gradually reduce the feature map’s output volume to prevent overfitting^46^. Max pooling and mean pooling are two methods used in the pooling layer to minimize data. While mean pooling computes the average value, max pooling selects the highest possible value.

1 $$f\left( z \right) = \left\{ \begin{gathered} z,\quad z > 0 \hfill \\ 0,\quad z \le 0 \hfill \\ \end{gathered} \right.$$

where *f*(*z*) is the output value of ReLU activation and z is a pixel image’s value. According to fully connected layer, it is the final layer in the multilayer perceptron architecture as shown in Fig. 3^47^. All neurons passed from the previous activation layer will be connected by this layer. At this point, neurons in the input layer must be flattened to be converted into one dimensional data^48^. After that, more than four classes can be classified using softmax activation that represents another version of the logistic regression process. The standard softmax function *σ*: *S*^*X*^ → (0,1)^*X*^, where *X* ≥ 1, takes vector *R* = (*r*_1_,…*r*_x_) *S*^*x*^ and computes each component of vector *σ*(*R*) $\in$ (0,1)^*X*^ using (2).

2 $${\upsigma }\left( {\text{R}} \right)_{i} = \frac{{e^{{r_{i} }} }}{{\mathop \sum \nolimits_{j = 1}^{x} e^{{r_{j} }} }}$$

where $\upsigma$ is softmax function, *R* is input vector, and ${e}^{{r}_{i}}$ is standard exponential function for input vector. *x* is number of classes in the multi-class classifier and ${e}^{{r}_{j}}$ is standard exponential function of output vector.

### Generative adversarial networks (GANs)

**Fig. 4:**
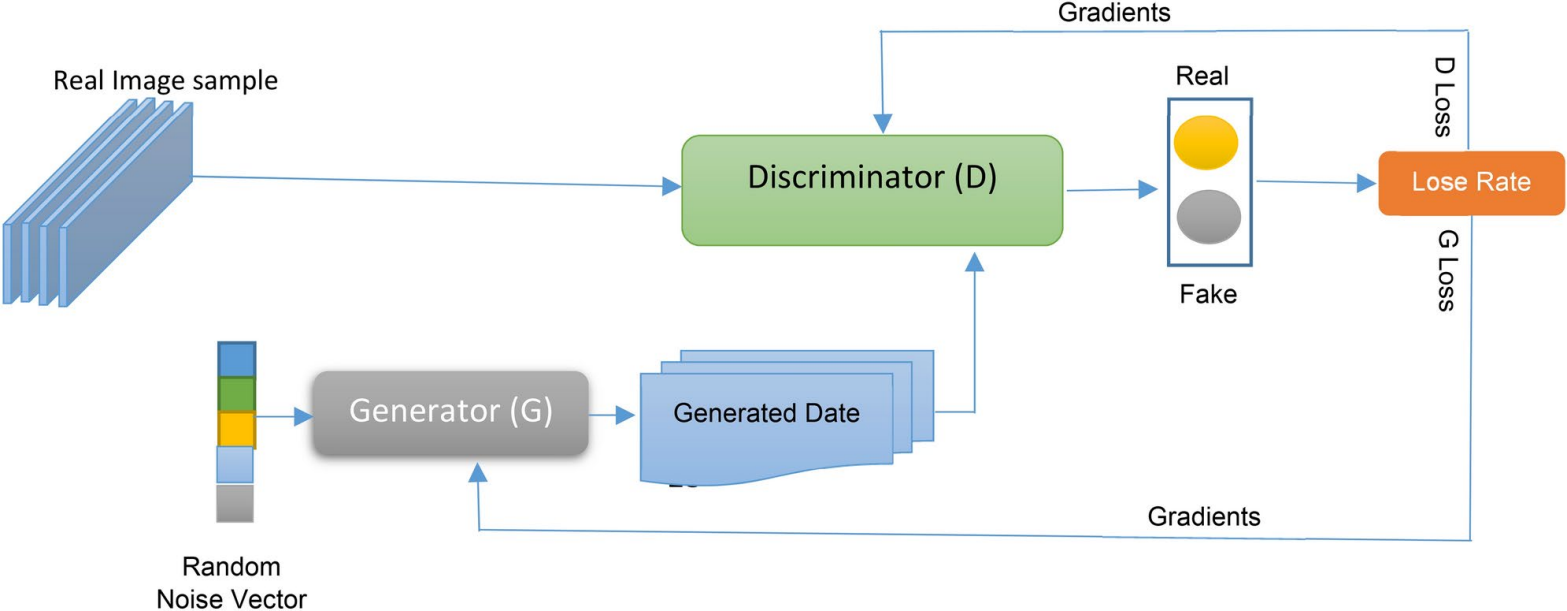
Generative adversarial network architecture.

In this section, a Generative Adversarial Network (GAN) will be used as an augmentation method, followed by a Convolutional Neural Network (CNN), which will be applied to the augmented data to diagnose bladder cancer patients. A GAN comprises two neural networks: the Generator (G) and the Discriminator (D). The Generator (G) learns to map points from a latent space to create synthetic images, while the Discriminator (D) is trained to differentiate between real images and those generated by G^49^. Over time, G improves its ability to create realistic images by receiving feedback from D, which simultaneously enhances its capability to differentiate real from fake images.^49^. The Discriminator’s loss function is based on the difference between real and fake labels, where fake images are generated by G, but the real label is assigned to them. Figure 3 illustrates the general architecture of GANs^49^. The primary objective of GAN theory is formulated as a two-player minimax game, which can be mathematically defined by Eq. (3)^49^ (Fig. 4).

3 $$Min_{G} Max_{D} V\left( {D,G} \right) = E_{A\sim Pd\left( A \right) } \left[ {\log D\left( A \right)} \right] + E_{s\sim Ps\left( s \right) } \left[ {\log \left( {1 - DG\left( s \right)} \right)} \right]$$

where the value function is *V*(*D*,*G*), the input data is *A*, the noise vector is *s*, the latent space’s probability distribution is *Ps* that is often a random Gaussian distribution, the training dataset’s probability distribution is *P*_*d*_, *D*(*G*(*s*)) is the discriminator’s output for a fake, *D*(*A*) is discriminator for *A* real, *E*_*A*~*Pd*_ is expectation data is real and *E*_*s*~*Ps*(*s*)_ is expectation data is fake.

**Fig. 5:**
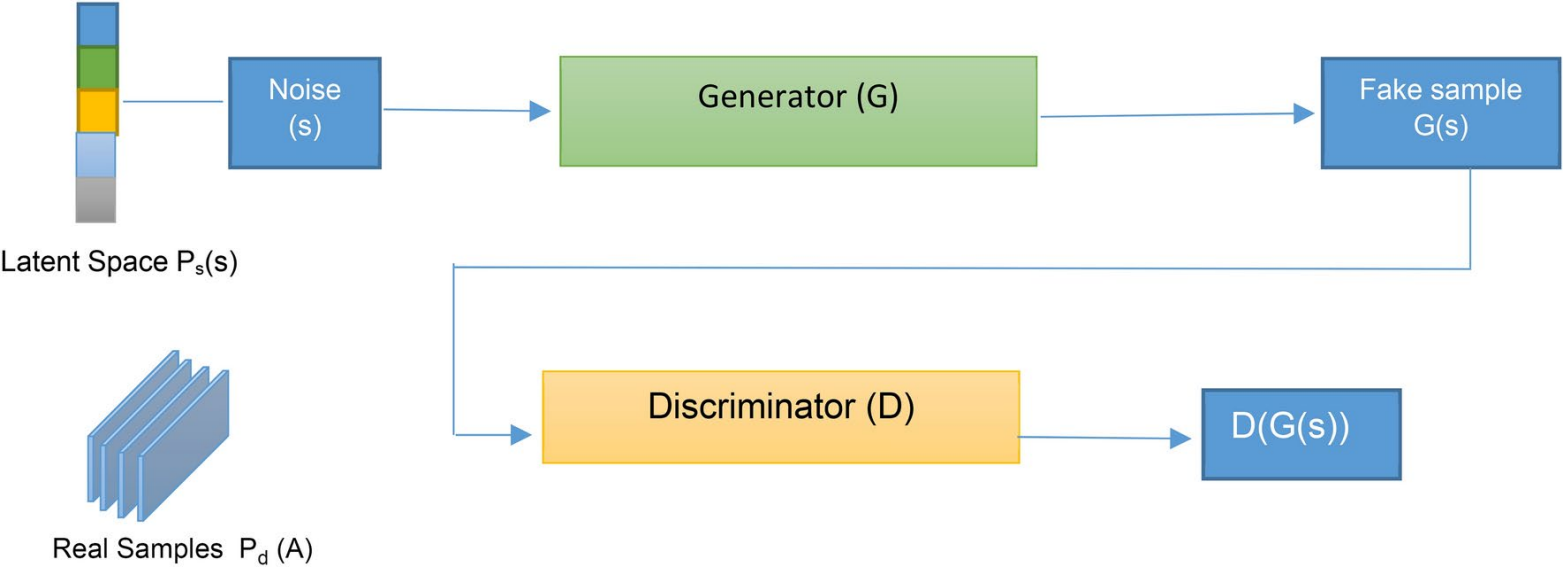
The objective function in GAN formulation.

According to G network, it creates synthetic samples with random noise (sampling from a latent space), while D network is a binary classifier that determines whether the input sample is real or fake (outputting a scalar value of 0). The samples produced by G are referred to as counterfeit samples. On the other hand, D detects a data point from the training dataset as a real sample when it uses this data as input, but it detects the other data point generated by G network as fake. According to D network, it wants to do its job in the best possible way. When a fake sample (which is generated by G) is given to D, it wants to call it out as fake, but G wants to generate samples in a way that makes D make a mistake in calling it out as a real one. A min–max optimization formulation will be used, in which D aims to maximize the same objective function, and G aims to minimize it, as shown in Fig. 5^49^. The main aim of D network is to reduce the probability of D(G(s)) to 0. Hence, it aims to maximize (1 − D(G(s))), while G network tries to make D mistakenly identify a created sample as real by forcing the likelihood of D(G(s)) to 1. Accordingly, G network aims to minimize (1 − D(G(s))). After implementing GAN, the augmented dataset will be passed to CNN for diagnosing bladder cancer cases.

### Explainable deep learning (XDL)

The traditional deep learning models excel in complex pattern recognition tasks, while explainable deep learning provides critical advantages in scenarios where understanding, trust, and regulatory compliance are paramount^50^. XDL generates more explainable models that maintain a high degree of learning performance (accuracy of predictions) and help humans’ comprehension, appropriate use, trust, and efficient management of the new breed of artificially intelligent companions. Thus, before deep learning techniques can be used in medical applications, it is essential to make them more interpretable. XDL models offer transparency in their decision-making process. Additionally, their ability to explain decisions enhances the trustworthiness of the model^51^. Users and stakeholders can verify the model’s decisions and understand its limitations and biases, leading to increased confidence in its use^51^.

The XDL applies a model called Gradient-weighted Class Activation Mapping (Grad-CAM) to evaluate and understand their choices. Grad-CAM detects the areas of the input image that contributed most to the final diagnose determination^52^. This aids in locating the crucial areas of The XDL applies a model called Gradient-weighted Class Activation Mapping (Grad-CAM) to evaluate and understand its choices. Grad-CAM detects the areas of the input image that contributed most to the final diagnosis determination^52^. This aids in locating the crucial areas of the image that the model relied on to make its predictions. Users may visually analyze and comprehend the model’s classification of a medical image into a certain category by superimposing the Grad-CAM heat map over the original image. Understanding the model’s logic and learning about its decision-making process depend on its interpretability. Interpretability and visual quality of the explanations are improved by combining the advantages of Guided Grad-CAM and guided backpropagation, which enhances both the interpretability and visual quality of the explanations. This research suggests an integrated neural network that depends on a Guided Gradient-weighted Class Activation Map (Guided Grad-CAM) as a visual attention-guided data augmentation strategy that targets the bladder cancer image dataset, aids in the model’s learning of rich, subtle characteristics, and increases identification accuracy.

**Fig. 6:**
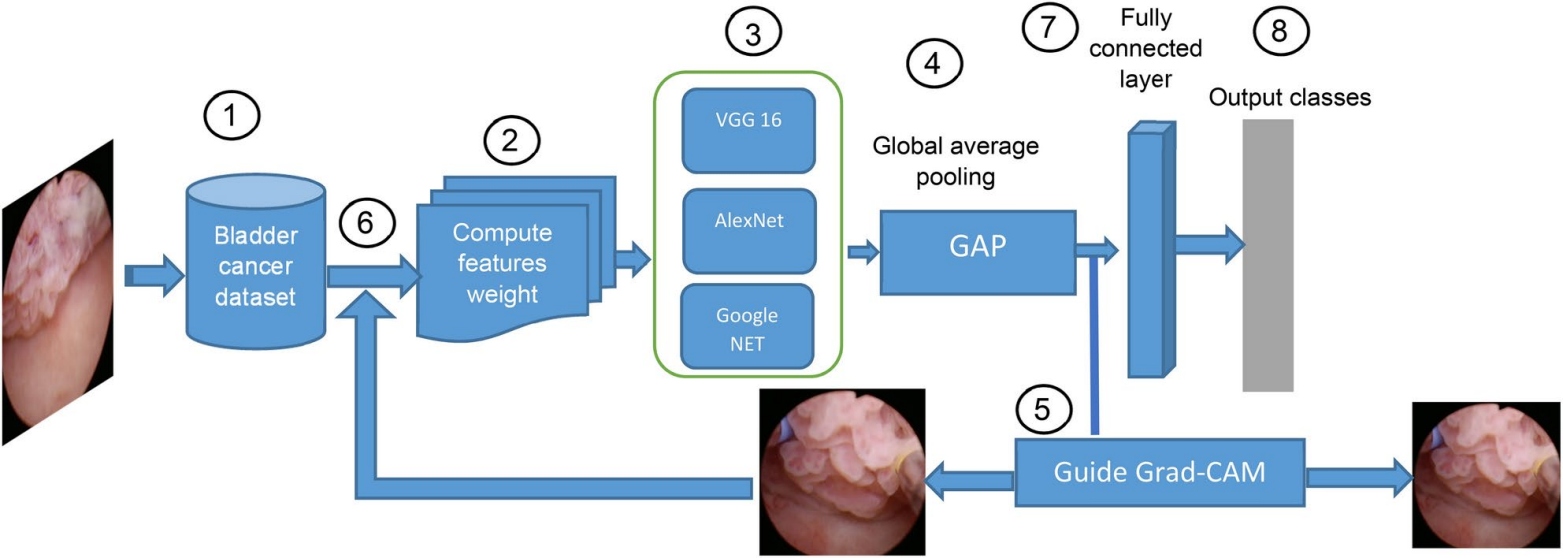
Explainable deep learning model.

The steps of the XDL model to accurately diagnose bladder cancer cases are displayed in Fig. 6. According to Fig. 5, in the first step, a bladder cancer image is input from the dataset to extract features and diagnose it. In the second step, it passes to VGG16 for extracting high-level features from the image. In the third step, the extracted features pass to Global Average Pooling (GAP), which lowers the overall training parameters and lowers the chance of overfitting. In the fourth step, the image passes to Guided Grad-CAM to improve and enhance images before classifying the disease. According to the fifth step, the image will be weighted using a new feature weight, and then this process will be repeated until the best feature weights are obtained. In the sixth step, the weighted features will pass through a fully connected layer to get the output classes.

**Fig. 7:**
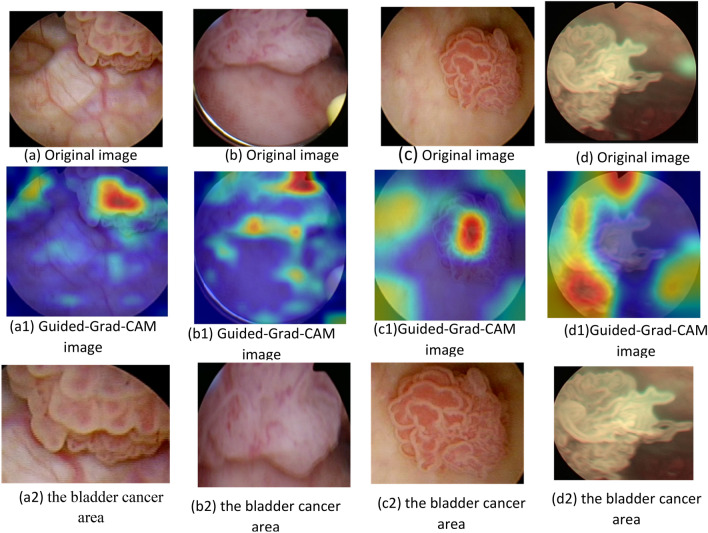
The enhancement of bladder cancer images.

According to the fourth step in Fig. 5, the main method in XDL is Guided Grad-CAM, which is used for enhancing the image and making it more explainable. The “Guided” in Guided Grad-CAM makes the images more accurate and improves the ability to accurately diagnose bladder cancer. It also helps deep learning (DL) to extract the characteristics more clearly and achieve a more accurate diagnosis of patients. The Guided Grad-CAM map is created using the feature map and the actual label after the convolutional network uses the training set as input to extract features and create a feature map. The source image is cropped to produce various training images, which are then entered into a convolutional network for training using the Guided Grad-CAM map’s attention mechanism. The final classification result is finally provided by the network output. A generic type of CAM that can be used with any convolutional deep learning method is called Grad-CAM^53^. Grad-CAM may often be calculated by selecting the final convolutional layer. Assume that the last convolutional layer’s output mapping is represented by the symbol *A*^*k*^. The final Grad-CAM (I^c^_Grad-CAM_) can be computed using (4)^52^.

4 $${\text{I}}^{{\text{c}}}_{{{\text{Grad}} - {\text{CAM}}}} = {\text{ReLU}}\left( {\mathop \sum \limits_{k = 1}^{K} w_{k}^{c} .A^{k} } \right)$$

where *ReLu* is an activation function used to map *A*^*k*^ in the weighted summation. *A*^*k*^ refers to input features of differential operations represented in W x H dimensions. *k* is an index for the total number of output maps (K). ${w}_{k}^{c}$ is produced for the class *c* and *Z* mapping *A*^*k*^ are normalization factors that are weighted. ${w}_{k}^{c}$ can be calculated using (5).

5 $$w_{k}^{c} = \frac{1}{z} \mathop \sum \limits_{i = 1}^{w} \mathop \sum \limits_{j = 1}^{H} \frac{{\partial y^{c} }}{{\partial A_{ij}^{k} }}$$

where ${w}_{k}^{c}$ are weights of output layers, y^c^ refers to the output of the class *C* depending on softmax. After adjusting the ReLu gradient backpropagation, the fraction below zero is not propagated, and only the fraction above 0 is propagated. Regarding results, when arriving at the first convolutional layer, the learned gradient used for further ReLu activation. At this stage, to display the gradients and identify the region of interest in the network, a new method of Grad-CAM called Guided Grad-CAM will be used for each prediction result. In fact, Guided Grad-CAM (${I}_{\text{Guided}-\text{Grad}-\text{CAM}}^{c}$) is calculated by multiplying the backpropagation and the separation activation map using (6). The final integrated classification result of Guided-Grad-CAM (${I}_{\text{Guided}-\text{Grad}-\text{CAM}}^{c}$) using normalization is presented in (7).

6 $$I_{{{\text{Guided}} - {\text{Grad}} - {\text{CAM}}}}^{c} = I_{{{\text{Guided}} - {\text{Backprop}}}}^{c} .I_{{{\text{Guided}} - {\text{CAM}}}}^{c}$$

7 $$I_{{{\text{Guided}} - {\text{Grad}} - {\text{CAM}}}}^{c} = \frac{1}{z} \mathop \sum \limits_{c = 1}^{c} I_{{{\text{Guided}} - {\text{Grad}} - {\text{CAM}}}}^{c}$$

where ${I}_{\text{Guided}-\text{Backprop}}^{c}$ is gradient backpropagation’s output and ${I}_{\text{Guided}-\text{CAM}}^{c}$ is the class activation map output. *Z* represents the normalization factor and *C* is the total number of categories classes which are referred to as c. Guided-Grad-CAM captures the most important regions of interest for a category, which was initially applied to CNN perception and aim of localization under weakly supervised conditions. Hence, Guided-Grad-CAM is used in this work to generate cropped images to guiding attention and obtain the local area of bladder images based on Guided-Grad-CAM. According to Fig. 7, the process of extracting the bladder cancer area in images by using the Guided-Grad-CAM algorithm will be explained. Figure 7a–d represents original images from dataset while Fig. 7a1–d1 represents the Guided-Grad-CAM images from the original bladder images. Additionally, Fig. 7a2–d2 are the superposition of both Fig. 7a–d and Fig. 7a1–d1. In other words, Fig. 7a2–d2 are the bladder cancer areas which are cut according to the position of red area in the Guided-Grad-CAM images. The improved images mentioned above are used as input into VGG16’s classification network.

**Fig. 8:**
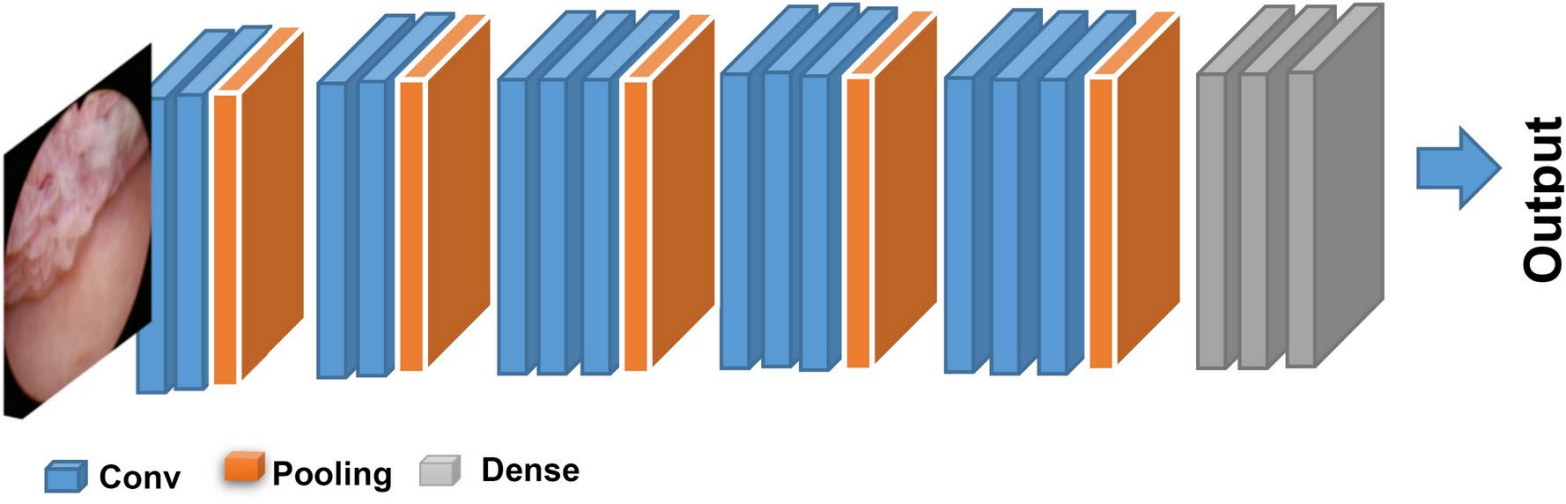
The architecture of VGG16 model.

According to the second step in Fig. 6, the three models (VGG16, AlexNet, and GoogleNet) were tested, and the best decision was made among them. Based on research, VGG16 was found to be the best. VGG16 is a deep learning model based on CNN architecture, which is used to interpret organized, grid-like data, including photographs^49^. It is made up of several layers, such as fully connected, pooling, and convolutional layers. CNNs’ hierarchical feature extraction capabilities make them highly useful for applications like object detection, image segmentation, and image classification. The CNN architecture, known as the VGG16 model, consists of 16 layers in total, including 13 convolutional layers and 3 fully connected layers. It is distinguished by its depth. VGG16 is widely recognized for its effectiveness and straightforward architecture, as well as for its strong performance across a variety of computer vision, applications such as object detection and image classification. As shown in Fig. 8, after a stack of convolutional layers and three fully-connected layers are placed, a Softmax layer is used as the last layer. These layers produce the class category results of bladder cancer disease.

### Voting method

In the proposed EDL, three types of deep learning classifiers were used. These classifiers are considered the best types of classifiers, as they provide high accuracy and precise results in the medical field. They also solve every issue that arose in the previous classifiers to reach the most accurate results. To obtain an accurate classification, the proposed EDL is applied and implemented. A majority voting method is used based on the output obtained from each classifier^54^. In this work, there are two main cases that will be followed. These cases are the normal case and the abnormal case. In the first case (normal), the same class is chosen by more than one classifier; therefore, this class is followed as a final result. On the other hand, in the second case (abnormal), the three classifiers give different class categories; thus, the evaluation is performed by calculating the accuracy value for each classifier, and the final result is determined by the most accurate classifier.

In the second case (abnormal), a main problem may appear if the difference in accuracy values between the classifiers is large; this leads to the Majority Fault Problem (MFP). MFP occurs when two classifiers may have low accuracy values and give the same class category, while the third classifier, which provides the maximum accuracy value, assigns a different class category. Although the third classifier is the most accurate one, the final decision is still determined by the other two less accurate classifiers since their combined accuracy values exceed that of the third classifier. Hence, the final decision may not be accurate. In this work, more accurate classifiers with similar accuracy values have been used to cooperate in order to provide more precise results. These classifiers are CNN, GAN, and XDL.

An illustrative example is presented in Table 2 to clarify the idea. It is assumed that there are three classifiers named CNN, GAN, and XDL, and three class categories labeled NST, HGC, and LGC. CNN, GAN, and XDL are assumed to have accuracy values of 78%, 82%, and 86%, respectively, where their values are close. If CNN assigns the NST class category, while GAN and XDL assign the HGC class category, the majority voting determines that the output is the HGC class category. On the other hand, if CNN assigns NST, GAN assigns LGC, and XDL assigns HGC, the final decision follows XDL, which has the highest accuracy value. Hence, the output is the HGC class category. The steps of the voting method are illustrated in Algorithm 1.

**Table 2:** Examples of majority voting.

Classifiers	CNN	GAN	XDL	Result
Accuracy	78%	82%	86%	
Case 1	NST	HGC	HGC	HGC
Case 2	NST	LGC	HGC	HGC

**Algorithm 1:**
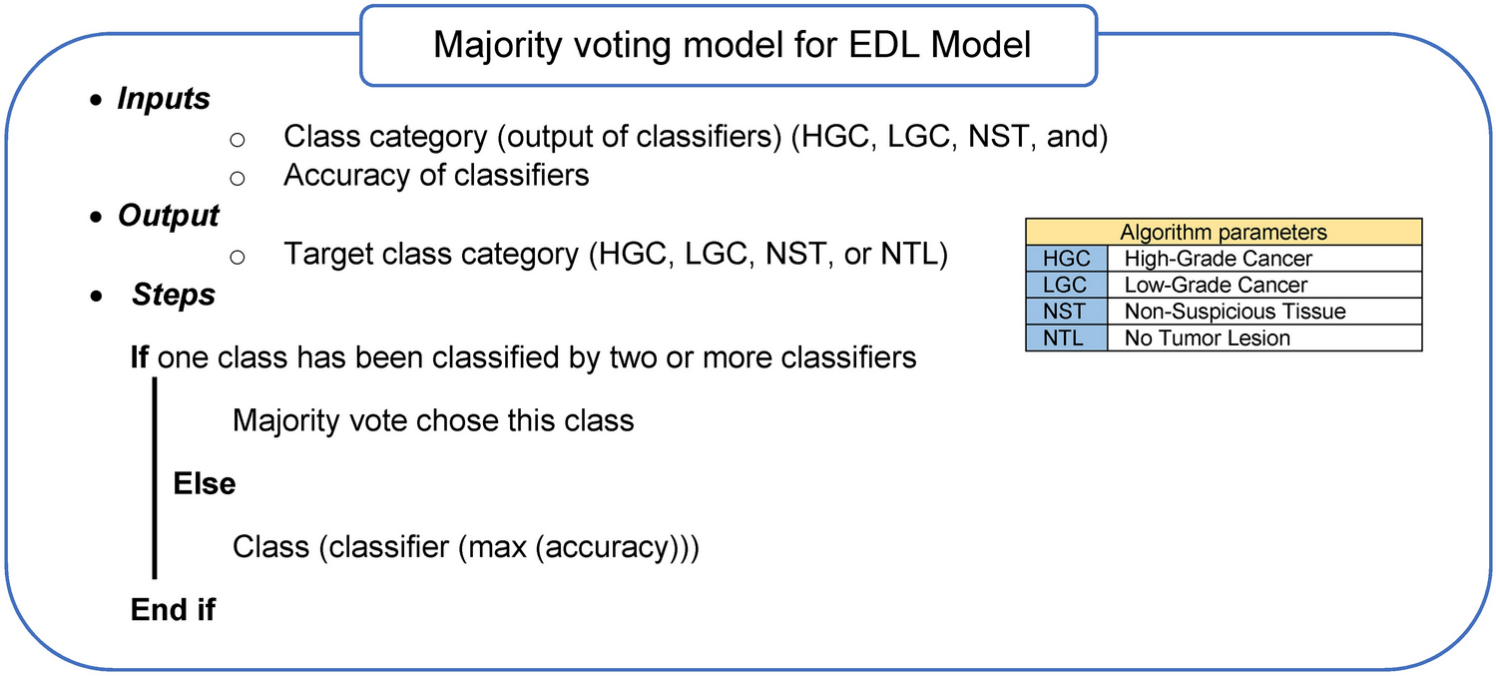
Majority voting model for EDL Model.

## Simulation and results

In this section, the proposed EDL model, which consists of three deep learning models: CNN, GAN, and XDL, will be implemented and compared against many modern diagnostic models to diagnose patients suffering from bladder cancer. To test the EDL, two main scenarios will be applied. The first scenario involves performing and testing the EDL against each deep learning model used in the construction of EDL namely CNN, GAN, and XDL as well as other deep learning models. The second scenario focuses on performing and testing the EDL model against other recent strategies used to diagnose bladder cancer. This ensures the effectiveness of the EDL model compared to other strategies. The dataset used is the Endoscopic Bladder Tissue dataset^19^. Hence, both scenarios for implementing the EDL model against other models will be performed using this dataset to prove that EDL is an accurate model capable of handling a wide variety of data as well as both large and small datasets. Several performance metrics such as precision, accuracy, recall, and error computed based on a confusion matrix are used to measure the performance of the models^11,12^. The values of the parameters used for CNN, GAN, and XDL are depicted in Table 5. The models were deployed on an Nvidia GeForce GTX 1080 GPU and created with Tensor Flow 2.5 in Python 3.6. Each of the several tests in this work included ten iterations of the classifiers’ training.

### Performance metric

Confusion matrix metrics are automated evaluation methods that categorize false positives, false negatives, true negatives, and true positives to assess the performance of diagnostic procedures. These metrics include accuracy, error, recall, precision, macro-average, micro-average, and F1-measure^11,12^. In this study, four primary evaluation metrics accuracy, recall, precision, and error are used to test the proposed EDL model in two different scenarios. Additionally, macro-average, micro-average, and F1-measure are included to further evaluate the performance of the EDL strategy compared to other strategies. Table 3 presents the confusion matrix, while Table 4 includes the formulas used to compute its metrics (Table 5).

**Table 3:** Confusion matrix.

		Classified label	
		Positive	Negative
Known label	Positive	True positive (TP)	False negative (FN)
	Negative	False positive (FP)	True negative (TN)

**Table 4:** Confusion matrix formulas.

Metric	Formula	Meaning
Precision (P)	${\text{TP}}/\left( {{\text{TP}} + {\text{FP}}} \right)$	The proportion of accurate positive forecasts
Recall (R)	${\text{TP}}/\left( {{\text{TP}} + {\text{FN}}} \right)$	The proportion of cases with positive labels that were expected to be positive
Accuracy	$\left( {{\text{TP}} + {\text{TN}}} \right)\left( {{\text{TP}} + {\text{TN}} + {\text{FP}} + {\text{FN}}} \right)$	The proportion of accurate forecasts
Error	${1} - {\text{Accuracy}}$	The proportion of forecasts that are wrong
Macro-average	$\mathop \sum \limits_{i = 1}^{c} p_{i} /c$ “for Precision”	The system’s average recall and accuracy across several classifications
	$\mathop \sum \limits_{i = 1}^{c} R_{i} /c$ “for Recall”	
Micro-average	$\left( {{\text{TP1}} + {\text{TP2}}} \right)/\left( {{\text{TP1}} + {\text{TP2}} + {\text{FP1}} + {\text{FP2}}} \right)$“for Precision”	The system’s total number of true positives, false positives, and false negatives for each class is added together and used to obtain the statistics
	$\left( {{\text{TP1}} + {\text{TP2}}} \right)/\left( {{\text{TP1}} + {\text{TP2}} + {\text{FN1}} + {\text{FN2}}} \right)$“for Recall”	
F1-measure	${2}*{\text{PR}}/\left( {{\text{P}} + {\text{R}}} \right)$	The precision and recall weighted harmonic mean

**Table 5:** The values of the used parameters.

Epochs	10 times		
	Layer	Input size	Output size
CNN parameters	Input	32 × 32 × 3	32 × 32 × 3
	Conv1	32 × 32 × 3	28 × 28 × 8
	Pool1	28 × 28 × 8	14 × 14 × 8
	Conv2	14 × 14 × 8	10 × 10 × 16
	Pool2	10 × 10 × 16	5 × 5 × 16
	Fully connected	5 × 5 × 16	48,120
	Fully connected	48,120	10,164
	Soft max	10,164	850
	Output	850	3 (classes)
XDL parameters	Layer	Input size	Output size
	Conv1-1-64	224 × 224 × 3	224 × 224 × 64
	Conv1-1-64	224 × 224 × 64	224 × 224 × 64
	Maxpool-1	224 × 224 × 64	112 × 112 × 64
	Conv2-1-128	112 × 112 × 64	112 × 112 × 128
	Conv2-1-128	112 × 112 × 128	112 × 112 × 128
	Maxpool-2	112 × 112 × 128	56 × 56 × 128
	Conv3-1-256	56 × 56 × 128	56 × 56 × 256
	Conv3-1-256	56 × 56 × 256	56 × 56 × 256
	Maxpool-3	56 × 56*256	28 × 28 × 256
	Conv4-1-512	28 × 28 × 256	28 × 28 × 512
	Conv4-1-512	28 × 28 × 512	28 × 28 × 512
	Maxpool-4	28 × 28 × 512	14 × 14 × 512
	Conv5-1-512	14 × 14 × 512	14 × 14 × 512
	Conv5-1-512	14 × 14 × 512	14 × 14 × 512
	Maxpool-5	14 × 14 × 512	7 × 7 × 512
	Fully connected	1 × 1 × 25,088	1 × 1 × 4096
	Fully connected	1 × 1 × 4096	1 × 1 × 4096
	Fully connected	1 × 1 × 4096	1 × 1 × 1000
	Output	1 × 1 × 1000	Soft max

### Description of bladder cancer dataset

**Fig. 9:**
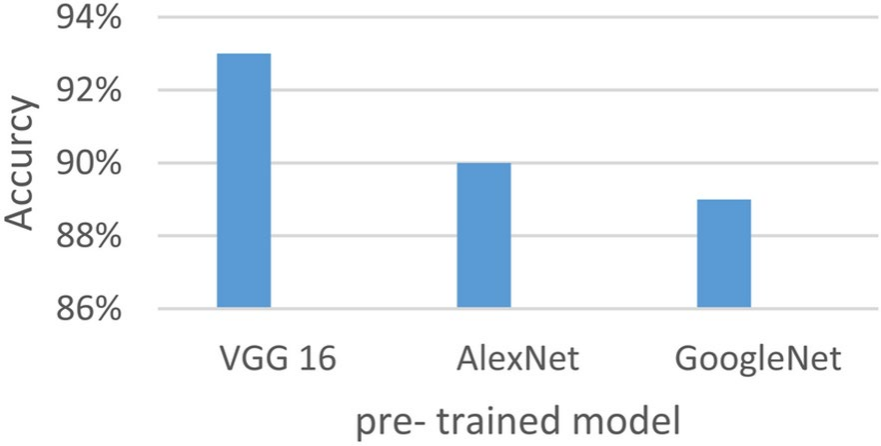
Accuracy of different deep leaning methods.

Endoscopic movies and corresponding histological analyses from resected lesions of 23 individuals undergoing TURBT were gathered for this study^31^. With the assistance of a professional surgeon, the visual data and histology results were matched by analyzing the videos frame by frame. The matching process was conducted to determine which bladder parts had lesions removed during the surgical procedure. To prevent ambiguity from multiple lesions of different types, only the frames containing individual lesions were included in the dataset. This process was applied to each White Light Imaging (WLI) video clip. Four classes categories were defined. Two categories were considered for malignant tissue, based on the general categorization of bladder cancer by the World Health Organization (WHO) and the International Society of Urological Pathology (ISUP)^51^. These categories are Low-Grade Cancer (LGC) and High-Grade Cancer (HGC). Additionally, two more categories were considered for Non-Suspicious Tissue (NST) and No Tumor Lesion (NTL), the latter encompassing cases of cystitis resulting from infections or other inflammatory factors. Table 6 displays the dataset’s comprehensive statistics. The videos were obtained from the European Institute of Oncology (IEO) in Milan, Italy. Every patient provided informed consent, complying with the Helsinki Declaration, and the study was approved by the IEO. No personal information was recorded. After outlier rejection is processed the data set become 1233 images. The dataset consists of 930 images for training, 101 images for testing, and 195 images for validation (Fig. 9).

**Table 6:** Composition pf the data set white light image (WLI).

Tissues types	No. of patient cases	No. of images for WLI
HGC	8	386
LGC	9	454
NST	5	439
NTL	5	97
total	23	1433

### Evaluating ensemble deep learning (EDL) against other deep learning methods

**Fig. 10:**
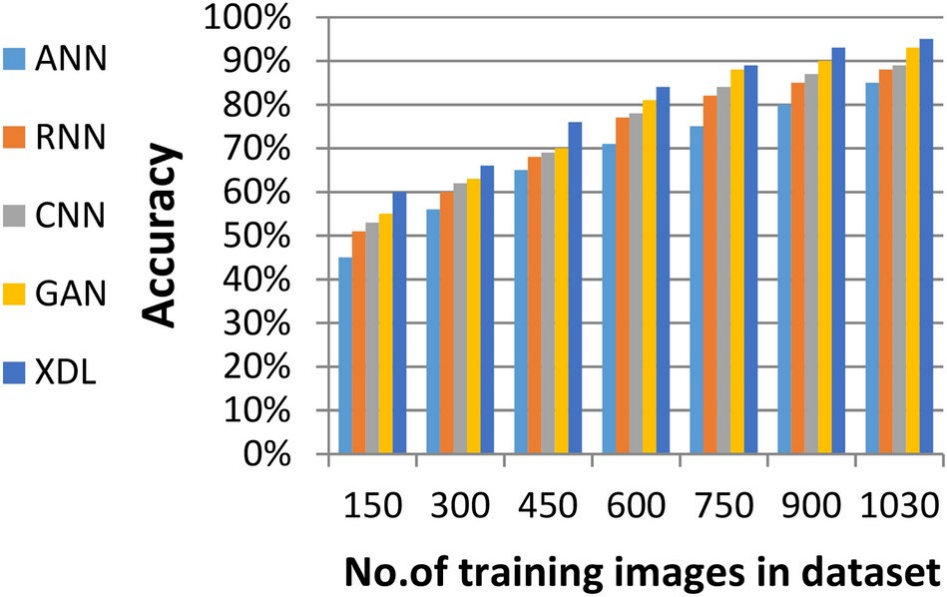
Accuracy of different deep leaning methods.

**Fig. 11:**
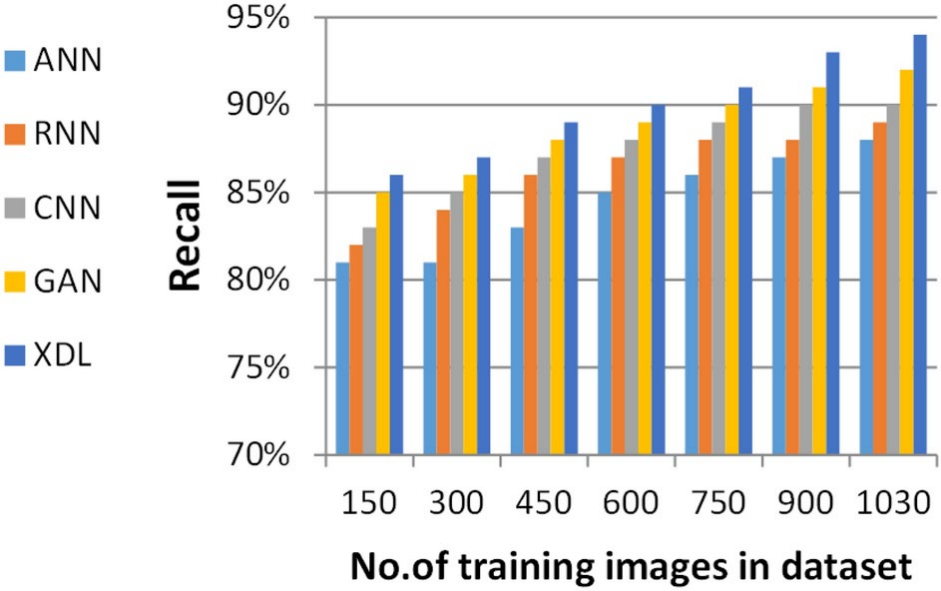
Recall of different deep leaning methods.

**Fig. 12:**
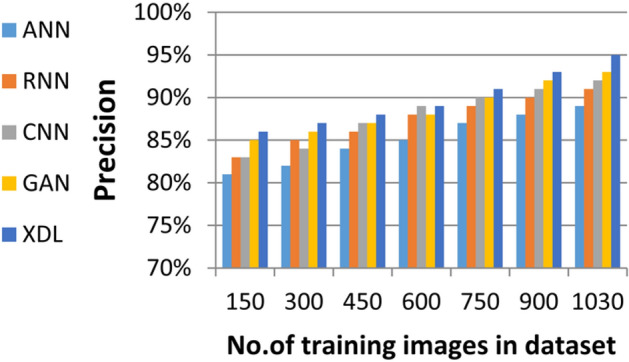
Precision of different deep leaning methods.

**Fig. 13:**
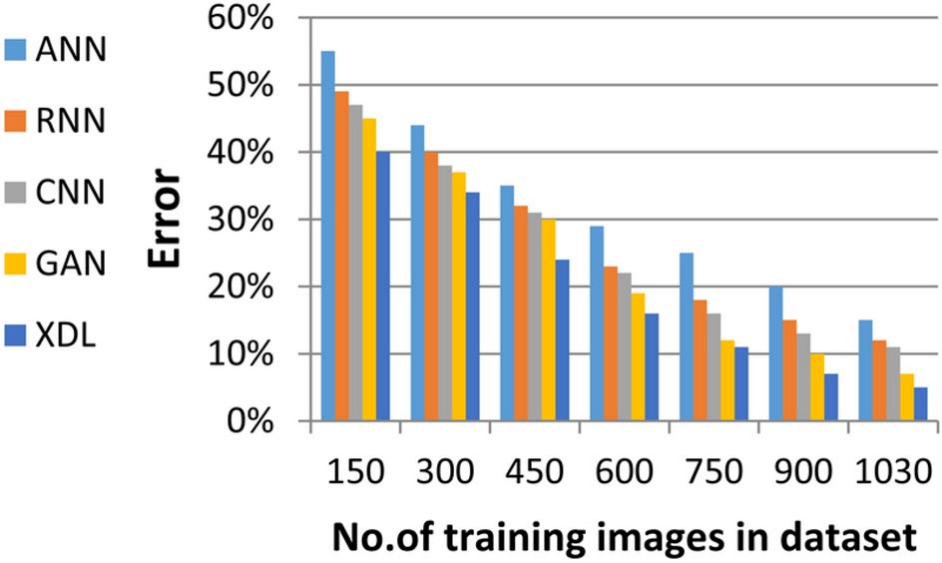
Error of different deep leaning methods.

**Fig. 14:**
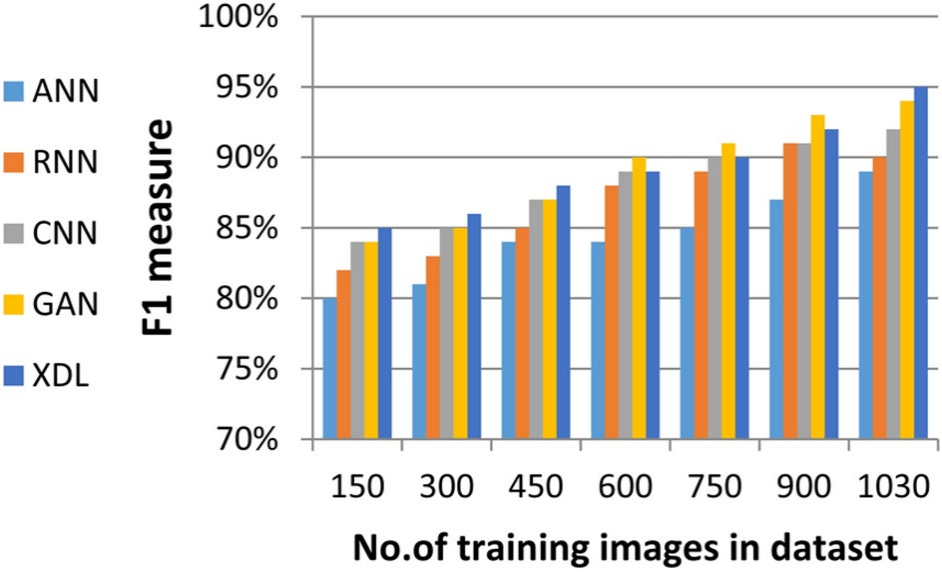
F1 measure of different deep leaning methods.

**Fig. 15:**
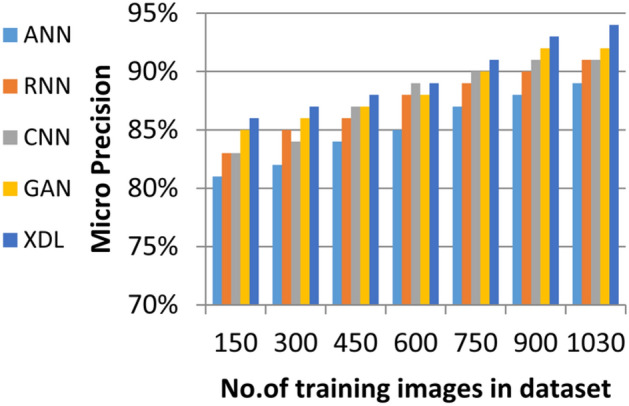
Micro Precision of different deep leaning methods.

**Fig. 16:**
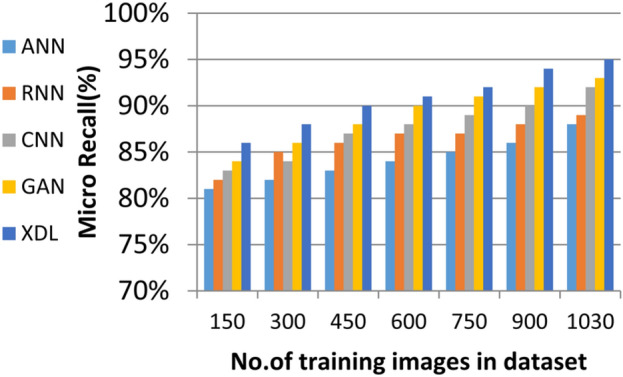
Micro Recall of different deep leaning methods.

**Fig. 17:**
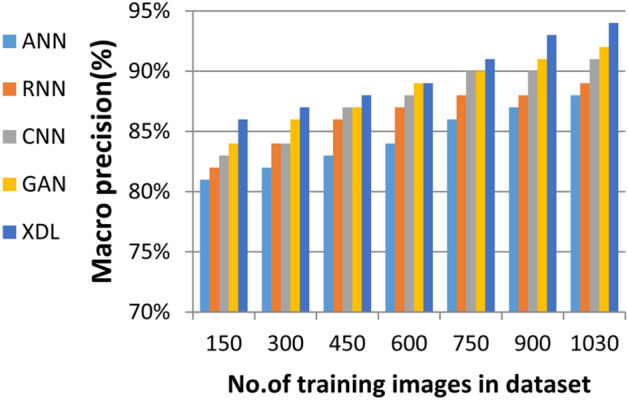
Macro Precision of different deep leaning methods.

**Fig. 18:**
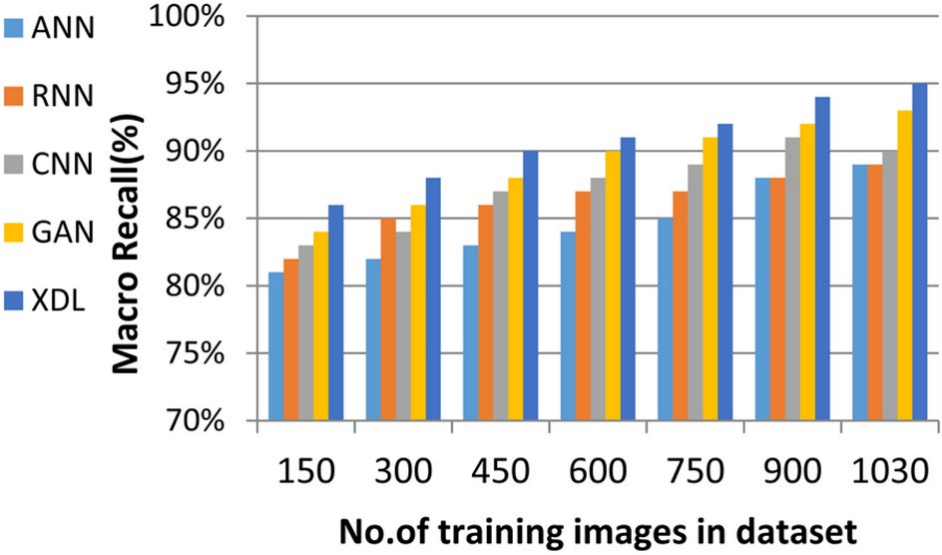
Macro Recall of different deep leaning methods.

**Fig. 19:**
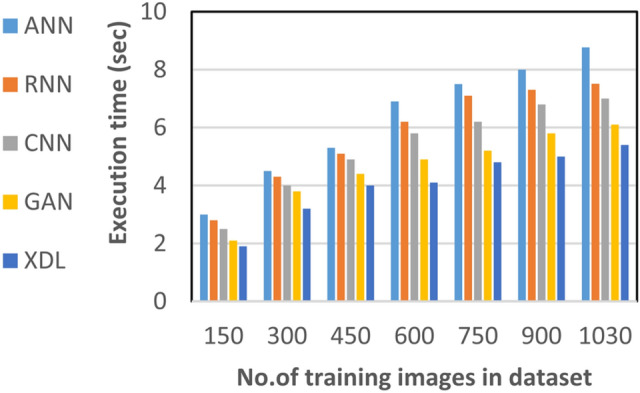
Execution of different deep leaning methods.

In this section, the proposed EDL model is evaluated against each deep learning model used in its construction, including CNN, GAN, and XDL, as well as other deep learning models such as Artificial Neural Network (ANN)^19^ and Recurrent Neural Network (RNN). Thus, the EDL model will be compared with ANN, RNN, CNN, GAN, and XDL. Table 7 and Figs. 10, 11, 12, 13, 14, 15, 16, 17, 18 and 19 present the results using confusion matrix metrics, including accuracy, recall, precision, error, F1-measure, macro-average, micro-average, and execution time. Also in the explainable pre-trained model compared VGG 16, AlexNet, GoogleNET and present the result in Table 8 and Figs. 10, 11, 12, 13, 14, 15, 16, 17, 18 and 19.

**Table 7:** The results of prediction methods at the maximum number of training samples.

Prediction Methods	ANN	RNN	CNN	GAN	XDL
Accuracy	85	88	89	94	95
Recall	88	89	89	93	94
Precision	89	91	92	94	94
Error	15	12	11	7	5
F1-measure	89	90	93	93	95
Macro precision	88	89	92	93	94
Micro precision	89	91	92	92	93
Macro recall	89	89	91	94	94
Micro recall	88	89	93	93	95
Execution time (s)	8.77	7.51	7	6.10	5.40

**Table 8:** Composition XDL accuracy for three pre- trained models.

Models	XDL accuracy
VGG 16	93%
AlexNet	90%
GoogleNet	89%

Figures 10, 11, 12, 13, 14, 15, 16, 17, 18 and 19 and Table 7 demonstrate that the deep learning models used in the proposed EDL model (CNN, GAN, and XDL) achieve the highest accuracy, recall, precision, F1-measure, and the lowest error compared to ANN and RNN when evaluated on the maximum number of training samples. Additionally, CNN, GAN, and XDL exhibit the lowest error and execution time relative to ANN and RNN under the same training conditions. According to Table 7 and Fig. 10, the accuracy of ANN, RNN, CNN, GAN, and XDL is 85%, 88%, 89%, 94%, and 95%, respectively. Similarly, Table 7 and Fig. 11 indicate that the recall values for ANN, RNN, CNN, GAN, and XDL are 88%, 89%, 89%, 93%, and 94%, respectively. As shown in Table 7 and Fig. 12, the precision values for ANN, RNN, CNN, GAN, and XDL are 89%, 91%, 92%, 94%, and 94%, respectively. Meanwhile, Table 7 and Fig. 13 highlight that the error rates for ANN, RNN, CNN, GAN, and XDL are 15%, 12%, 11%, 7%, and 5%, respectively. Finally, Table 7 and Fig. 14 illustrate that the F1-measure for ANN, RNN, CNN, GAN, and XDL is 89%, 90%, 93%, 93%, and 95%, respectively.

Figures 15, 16, 17, 18 and Table 7 demonstrate that micro precision, micro recall, macro precision, and macro recall improve as the number of training samples increases. According to Figs. 15, 16, and Table 7: ANN achieves 89% for micro precision and 88% for micro recall. RNN achieves 91% for micro precision and 89% for micro recall. CNN achieves 92% for micro precision and 92% for micro recall. GAN achieves 93% for micro precision and 92% for micro recall. XDL achieves the highest values, with 94% for micro precision and 93% for micro recall.

Figures 17, 18, and Table 7 show that ANN presents about 88% and 89% for macro precision and macro Recall respectively at the maximum number of training samples. RNN presents about 89% and 89% for macro precision and macro recall respectively at the maximum training sample number. Also, CNN presents about 92% and 91% for macro precision and macro recall respectively and GAN presents about 93% and 94% for macro precision and macro recall respectively. Finally, XDL presents 94% and 94% for macro precision and macro recall respectively at the same number of training samples. According to Fig. 19 and Table 7 the maximum execution time is provided by ANN and RNN but CNN, GAN, and XDL models provided the minimum execution time. The execution time of ANN and RNN are 8.77s and 7.51s respectively at the maximum number of training. On the other hand, the execution time of CNN, GAN, and XDL are 7s, 6.1s and 5.4s respectively.

From previous results in Figs. 10, 11, 12, 13, 14, 15, 16, 17, 18 and 19 and Table 7, it is noted that CNN, GAN, and XDL provide more accurate diagnosis at the least execution time compared to ANN and RNN. In fact, CNN, GAN, and XDL methods provide the highest and closed accuracy values, therefore, these deep learning methods were chosen to cooperate and construct the proposed EDL method. The proposed EDL will be tested and compared against other models in the next subsection.

### Evaluating ensemble deep learning (EDL) against other diagnoses strategies

**Fig. 20:**
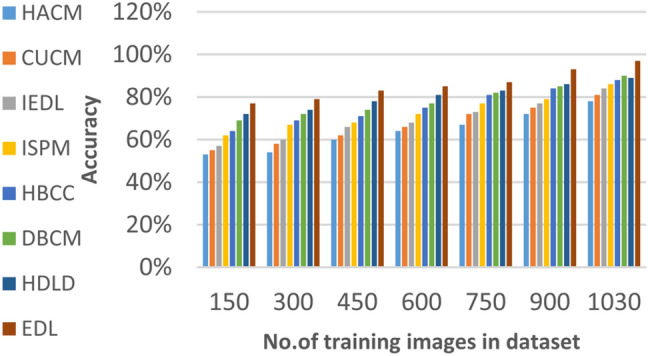
Accuracy of bladder cancer models.

**Fig. 21:**
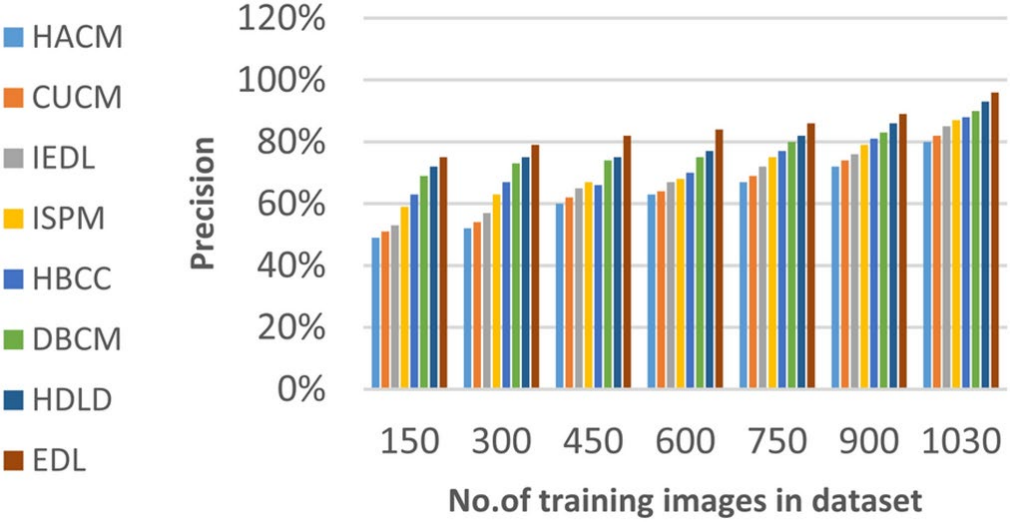
Precision of bladder cancer models.

**Fig. 22:**
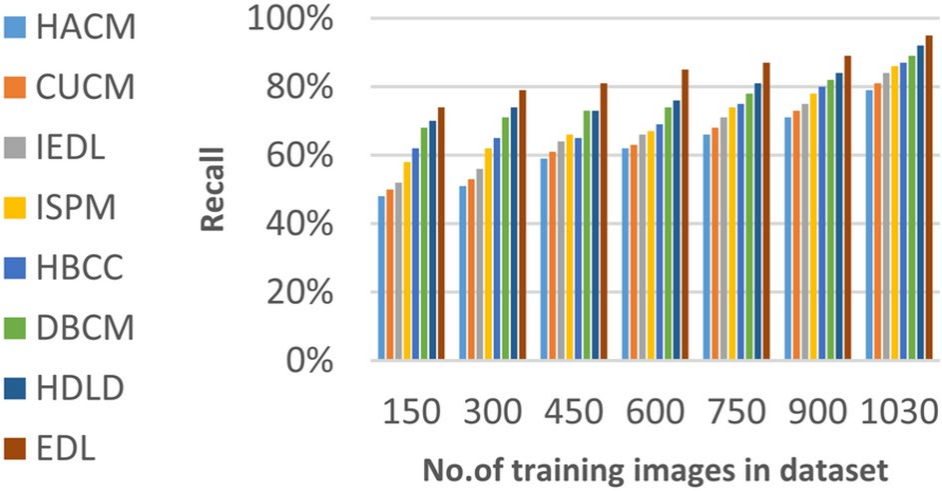
Recall of bladder cancer models of different deep leaning methods.

**Fig. 23:**
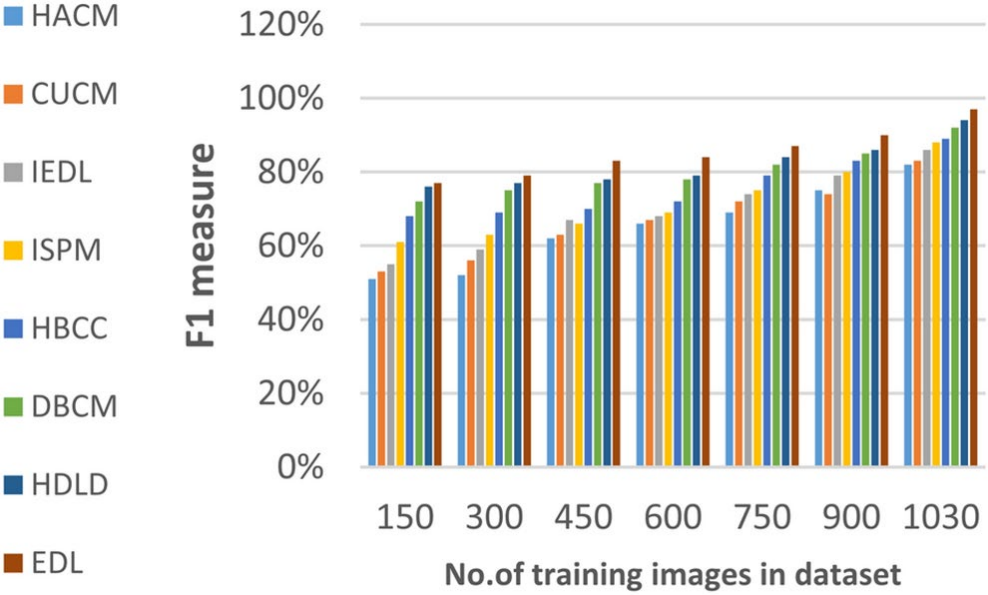
F1 measure of bladder cancer models of different deep leaning methods.

**Fig. 24:**
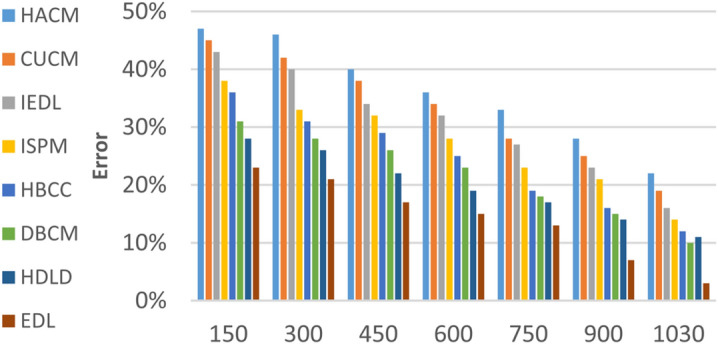
Error of bladder cancer models of different deep leaning methods.

**Fig. 25:**
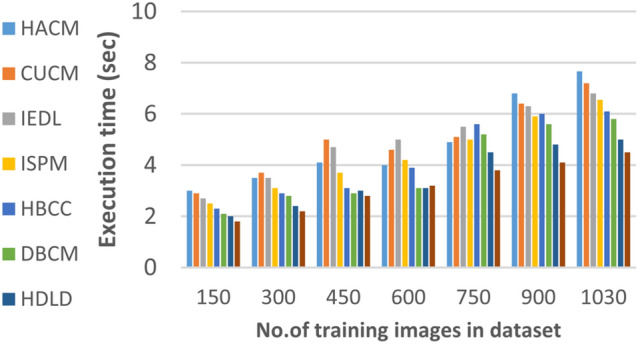
Execution of bladder cancer models of different deep leaning methods.

**Fig. 26:**
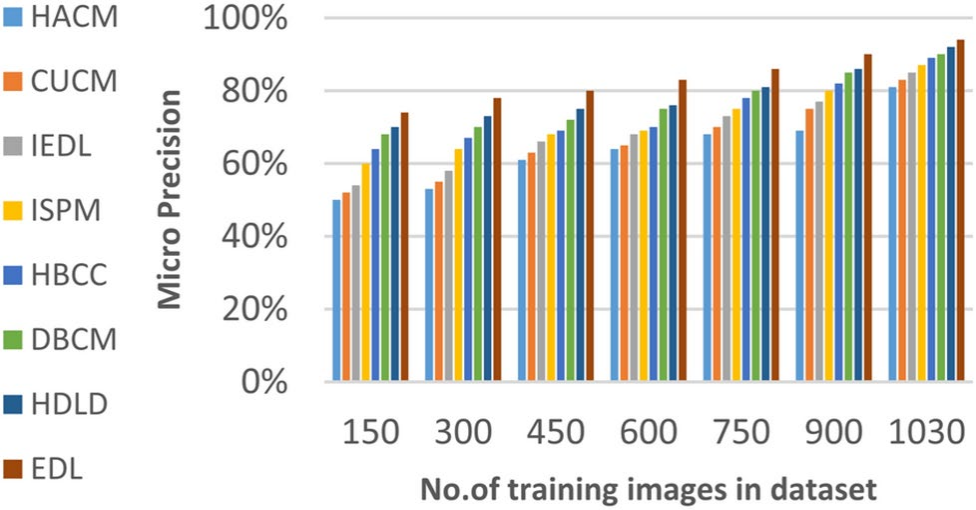
Micro Precision of bladder cancer models of different deep leaning methods.

In this section, the proposed EDL model will be tested to ensure its effectiveness compared to other deep learning diagnoses strategies. These strategies are HACM^24^, CUCM^25^, IEDL^26^, ISPM^27^, HBCC^28^, DBCM^29^ and HDLD^30^ which are presented in Table 1. Figures 20, 21, 22, 23, 24, 25, 26, 17, 28 and 29 and Table 6 illustrate the accuracy, error, precision, recall, macro average, micro average, F1-measure and execution time of EDL against other strategies. Figure 25 and Table 9 show that EDL model can provide accurate results in the minimum execution time.

**Fig. 27:**
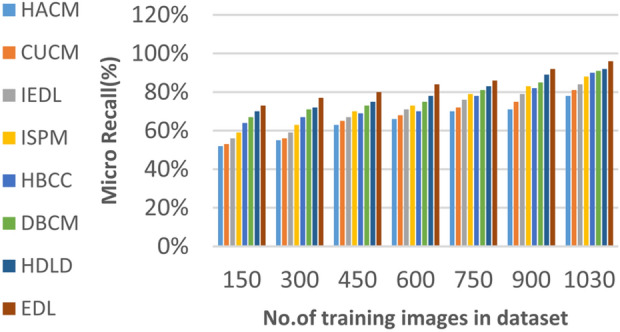
Micro Recall of bladder cancer models of different deep leaning methods.

**Fig. 28:**
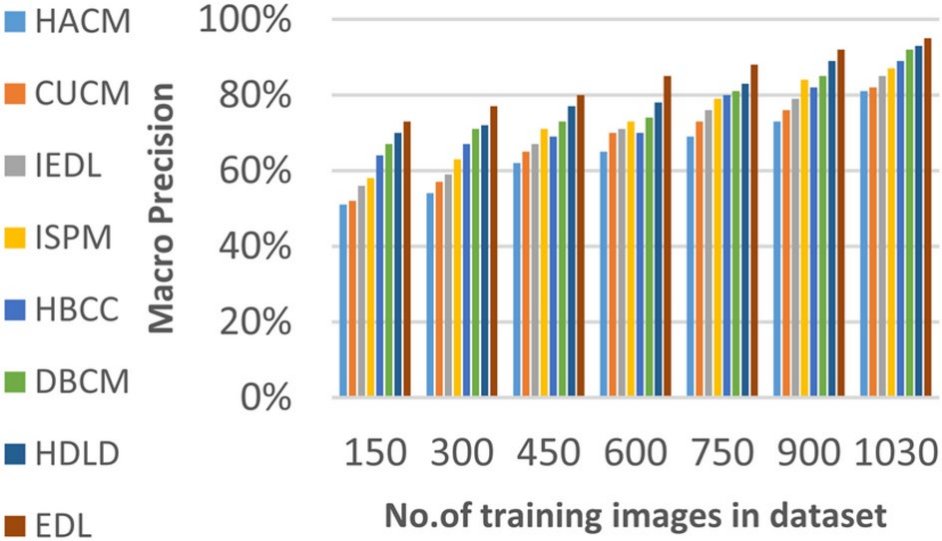
Macro Precision of bladder cancer models of different deep leaning methods.

**Fig. 29:**
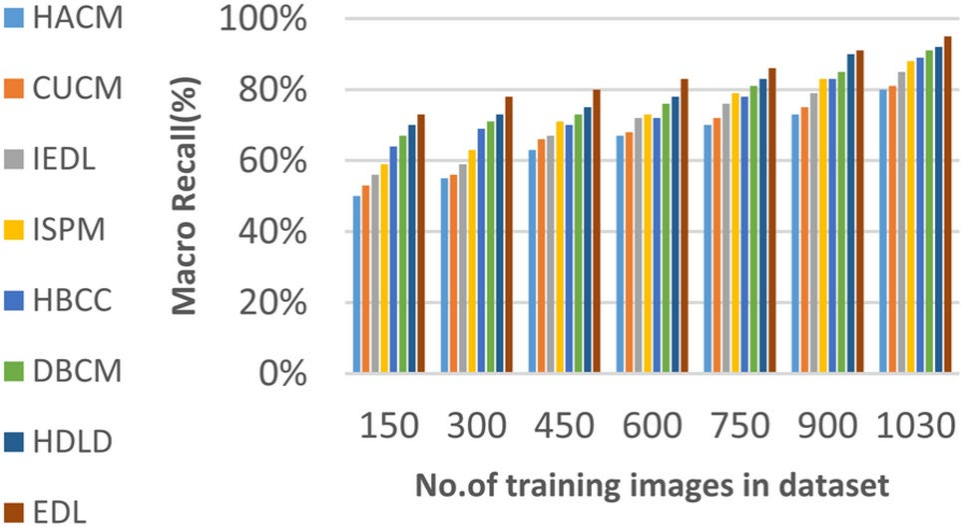
Macro Recall of bladder cancer models of different deep leaning methods.

**Table 9:** The results of EDL model against other methods at the maximum number of training samples.

Prediction Methods	HACM	CUCM	IEDL	ISPM	HBCC	DBCM	HDLD	EDL
Accuracy	78	81	84	86	88	90	93	98
Recall	79	81	84	86	87	89	92	97
Precision	80	82	85	87	88	90	93	96
Error	22	19	16	14	12	10	11	3
Macro precision	81	82	85	87	89	92	93	97
Micro precision	81	83	85	87	89	90	92	96
Micro recall	78	81	84	88	90	91	92	96
Macro recall	80	81	85	88	89	91	92	97
F1-measure	82	83	86	88	89	92	94	95
Execution time (s)	7.66	7.2	6.8	6.55	6.1	5.8	5	5

Figures 20, 21, 22, 23, 24 and 25 and Table 9 show that EDL model provides the maximum accuracy, precision, recall, and F1-measure but the minimum error and execution time values compared to other models according to the maximum number of training samples. Related to Fig. 18 and Table 8, the accuracy of HACM, CUCM, IEDL, ISPM, HBCC,DBCM, HDLD, and EDL are 78%, 81%, 84%, 86%, 88%, 90%, 93%, and 98% respectively at the maximum number of training samples. Figure 19 and Table 8 show that the precision of HACM, CUCM, IEDL, ISPM, HBCC, DBCM, HDLD, and EDL are 80%, 82%, 85%, 87%, 88%, 90%, 93%, and 97% respectively.

According to Fig. 22 and Table 9, the recall of HACM, CUCM, IEDL, ISPM, HBCC, DBCM, HDLD, and EDL are 79%, 81%, 84%, 86%, 87%, 89%, 92%, and 96% respectively at the maximum number of training samples. According to Fig. 23 and Table 9, F1-measures of HACM, CUCM, IEDL, ISPM, HBCC,DBCM, HDLD, and EDL are 82%, 83%, 86%, 88%, 89%, 92%, 94%, and 95% respectively at the maximum number of training samples. Figure 24 and Table 9 show that the error of HACM, CUCM, IEDL, ISPM, HBCC, DBCM, HDLD, and EDL are 22%, 19%, 16%, 14%, 12%, 10%, 11%, and 3% respectively. According to Fig. 25 and Table 9, the maximum execution time is provided by HACM, CUCM, IEDL, ISPM, HBCC, DBCM, and HDLD but EDL model gives the minimum execution time at the maximum training sample number. The execution time of HACM, CUCM, IEDL, ISPM, HBCC, DBCM, HDLD, and EDL are 7.66s, 7.2s, 6.8s, 6.55s, 6.1, 5.8s, 5s and 5s respectively.

Figures 26, 27, 28, 29 and Table 9 show that the performance of micro precision, micro recall, macro precision, and macro recall are increasing according to the number of training samples. According to the maximum number of training samples, Figs. 26, 27 and Table 9 show that HACM introduces 81% and 78% for micro precision and micro recall respectively. Additionally, CUCM introduces 83% and 81% for micro precision and micro recall respectively. IEDL introduces 85% and 84% for micro precision, micro recall respectively. ISPM introduces 87% and 88% for micro precision and micro recall respectively. HBCC introduces 89% and 90% for micro precision and micro recall respectively. DBCM introduces 90% and 91% while HDLD introduces 92% and 92% for micro precision, micro recall respectively. EDL introduces 96% and 96% for micro precision and micro recall respectively. Thus, EDL outperforms other models according to micro precision and micro recall.

From Figs. 28, 29 and Table 9, HACM presents 81% and 80% for macro precision and macro recall respectively at the maximum number of training samples. CUCM presents 82% and 81% for macro precision and macro recall respectively at the maximum number of training samples. IEDL presents 85% and 85% while ISPM presents 87% and 88% for macro precision and macro recall respectively at the maximum number of training samples. HBCC presents 89% and 89% for macro precision and macro recall while DBCM presents 92% and 91% respectively at the maximum number of training samples. HDLD presents 93% and 92% for macro precision and macro recall respectively at the maximum number of training samples. Finally, EDL presents 97% and 97% for macro precision and macro Recall respectively at the maximum training sample number.

From previous results in Figs. 20, 21, 22, 23, 24, 25, 26, 27, 28, 29 and Table 9, it is noted that EDL provides more accurate diagnosis at the least execution time compared to HACM, CUCM, IEDL, ISPM, HBCC, DBCM, and HDLD. Additionally, EDL gives the maximum recall, precision, F1-measure, micro precision, micro recall, macro precision, and macro recall while it gives the minimum error and execution time values. Thus, the EDL model has proven its efficiency for providing an accurate diagnosis for bladder cancer cases according to a large and diverse dataset.

**Fig. 30:**
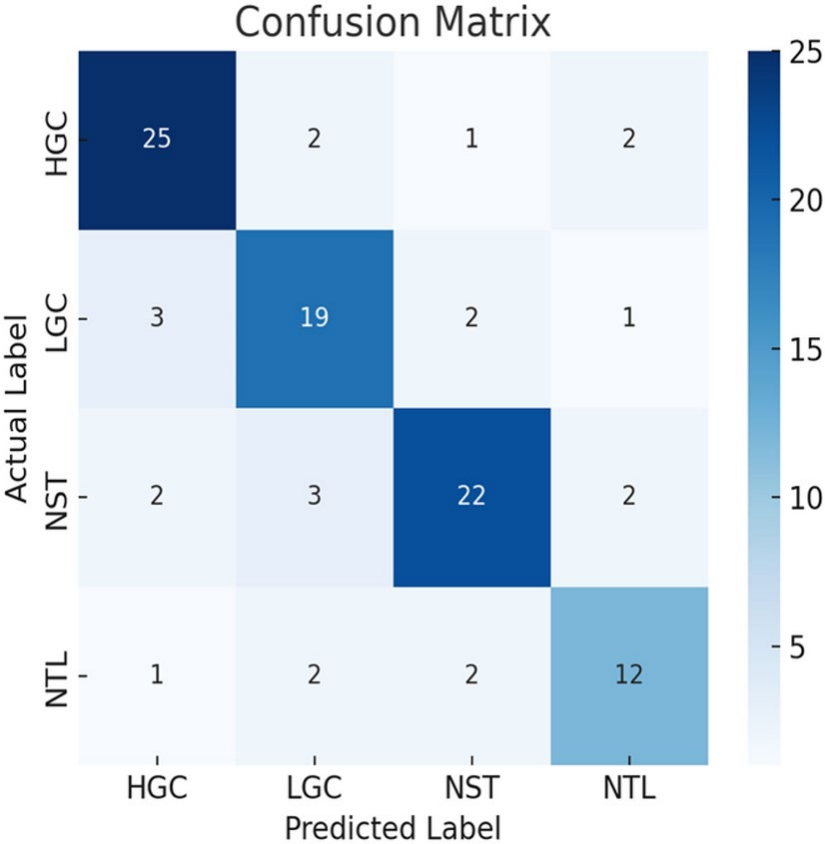
Confusion matrix.

Finally, the ablation investigations in Table 10 demonstrate how ensemble classifiers collaborate to enhance performance, confirming the effectiveness of EDL. The confusion matrix generated by the proposed EDL model is shown in Fig. 30. This illustrates EDL’s power and suitability for use with deep learning models to diagnose bladder cancer and achieve optimal outcomes.

**Table 10:** Ablation studies of key components of the proposed EDL model.

CNN	GAN	XDL	Accuracy
√			89%
	√		94%
		√	95%
√	√		96%
√		√	95%
	√	√	96%
√	√	√	98%

## Pros and cons

The benefits and cons of the EDL approach will be discussed in this part, as indicated in Table 11. EDL is a unique, adaptable, accurate, efficient, and scalable technique that offers several advantages over previous models. EDL is also capable of handling datasets that are not balanced. The EDL model actually has a high efficiency since it filters images using outlier rejection and then integrates three techniques: CNN, GAN, and XDL. As seen in Table 11. Despite the advantages of the EDL Model, its speed is slow and its complexity is considerable. However, as compared to producing correct results, the EDL model’s execution time is insignificant. Additionally, feature selection and outlier rejection may be done offline, thus the execution time has no impact on the system performance.

**Table 11:** The EDL pros and cons.

Pros	Cons		
Feature	Description	Feature	Description
Accuracy	EDL demonstrated higher diagnostic accuracy in comparison to other recent approaches when applied to the dataset	Delay	EDL requires a lot of time to execute because it combines three models
Scalability	EDL can deal with large dataset		
Applicability	EDL can be used in hospitals and medical facilities to lessen the workload for both patients and medical personnel		
Effectiveness	EDL can efficiently handle a real problem	Complexity	EDL is a complicated method that incorporates a number of complicated techniques
Robustness	For a precise diagnosis of bladder cancer, EDL robustness is maintained by data diversity, model tuning, noise resilience, and ongoing assessment		

## Conclusions and future works

In this paper, a new bladder cancer diagnosis model called Ensemble Deep Leaning (EDL) model has been introduced to present fast and accurate results. Outlier rejection used to filter data from dataset. EDL model consists of three model, which are; Convolutional Neural Network (CNN), Generative Adversarial Network (GAN), and Explainable Deep Learning (XDL) as a new model. XDL used new strategy called Gradient Grid-CAM which enhances images and make model more explainable. In EDL, the results of CNN, GAN, and XDL have been combined by using new voting strategy that depends on majority voting if all classifiers give the same class category. In the case if CNN, GAN, and XDL give different class categories, thus, the final result based on the accuracy of each class. According to experimental results, EDL proved it’s effectively as it can give the highest accuracy values. Additionally it can give the highest precision, and recall values on the other hand it give the lowest error value and execution time compared to other models with values equal to 98%, 97%, 96%, 3%, and 5s respectively at the maximum number of training samples. Finally, the proposed EDL model can present fast and accurate predictions. In the future, the proposed EDL model will be improved by using other deep learning algorithms that can provide more accurate results. Also, the data will be preprocessed and resized before using the classification methods.

### Experimental protocols

All experimental protocols were approved by Mansoura University, Faculty of Engineering.

## Data Availability

Dataset is available at: https://www.kaggle.com/datasets/aryashah2k/endoscopic-bladder-tissue-classification-dataset?resource=download.
